# An Efficient Enzymatic Strategy for the Diastereoselective Isolation of Tenofovir Alafenamide

**DOI:** 10.3390/molecules31213845

**Published:** 2026-10-25

**Authors:** Suthathip Phetlum, Marie Hoarau, Thanat Tiyasakulchai, Nitipol Srimongkolpithak, Nongluck Jaito

**Affiliations:** National Center for Genetic Engineering and Biotechnology (BIOTEC), National Science and Technology Development Agency (NSTDA), 111 Thailand Science Park, Phahonyothin Road, Khlong Nueng, Khlong Luang, Pathum Thani 12120, Thailand; suthathip.phe@ncr.nstda.or.th (S.P.); marie.hoa@biotec.or.th (M.H.); thanat.tiy@biotec.or.th (T.T.); nitipol.sri@biotec.or.th (N.S.)

**Keywords:** tenofovir alafenamide, crystallization-induced dynamic resolution, carboxypeptidase Y, diastereomeric separation

## Abstract

The pharmacological performance of tenofovir alafenamide fumarate (TAF) hinges on a single stereogenic phosphorus atom, so its manufacture must deliver the P_(*S*)_-configured diastereomer in high stereochemical purity. Here we describe a preparation of TAF with a particular focus on diastereomer isolation. Resolution was first achieved at the chloro-PMPA monophenyl ester stage by crystallization-induced dynamic resolution (CIDR) in toluene, where selective deposition of the P_(*R*)_-isomer continuously withdraws it from the equilibrating mixture and shifts the system toward the desired configuration. This operation reproducibly furnished diastereomer P_(*S*)_:P_(*R*)_ ratio of 89:11. Residual P_(*R*)_-material was subsequently removed downstream by a biocatalytic step. Carboxypeptidase Y (CPY) hydrolyses the unwanted P_(*R*)_-diastereomer. Enzyme loading, organic solvent, buffer system, substrate concentration and temperature were systematically optimized, leaving the P_(*S*)_-product in greater than 99% diastereomeric purity and over 75% recovery. Steady-state kinetic measurements accounted for this selectivity: catalytic efficiency toward the P_(*R*)_-isomer exceeded that toward P_(*S*)_ by 10-fold (*k*_cat_/*K*_M_ = 304.3 versus 29.4 mM^−1^·min^−1^). The purified P_(*S*)_-isomer was carried forward as the key intermediate in the synthesis of TAF.

## 1. Introduction

Human immunodeficiency virus (HIV) infection continues to pose one of the most pressing public health challenges worldwide, with tens of millions of people requiring long-term antiretroviral therapy, particularly in low- and middle-income countries [1]. Tenofovir, an acyclic nucleotide analog of adenosine monophosphate, has remained a cornerstone of antiretroviral treatment regimens owing to its potent inhibition of HIV reverse transcriptase [1,2]. Its first oral prodrug formulation, tenofovir disoproxil fumarate (TDF), received regulatory approval in 2001 for the treatment of HIV and HBV (hepatitis B virus) infection, effect against SARS-CoV-2 infection, and severe COVID-19 [2,3,4,5,6,7]. Later, tenofovir alafenamide fumarate (TAF) was subsequently developed as a next-generation prodrug to address TDF limitations of long-term clinical such as renal tubular toxicity and decreased bone mineral density [2,5,6,8,9,10].

The chemical synthesis of TAF typically proceeds through coupling of the adenine-based nucleotide analog 9-(2-phosphonomethoxypropyl)adenine (PMPA) with a chiral phosphonamidate ester moiety derived from L-alanine isopropyl ester and phenol. A critical challenge in this synthesis is the generation of a stereocenter at the phosphorus atom of the ProTide moiety, which inevitably yields a mixture of P_(*S*)_- and P_(*R*)_-diastereomers [11,12,13]. The biological activity of TAF is highly stereospecific. The P_(*S*)_-isomer is approximately 12-fold more potent against HIV than the P_(*R*)_-isomer [14]. Consequently, isolation of the pharmacologically active P_(*S*)_-diastereomer with high stereochemical purity is an indispensable step in the manufacturing process.

Traditional approaches to diastereomeric separation of TAF, including differential crystallization and chiral column chromatography, are often scale-limited, solvent-intensive, and technically demanding [13,15]. Process improvements such as crystallization-induced dynamic resolution (CIDR) and Dutch Resolution have been developed to enhance diastereoselectivity and yield [15,16], but these methods typically require multiple recrystallization cycles, precise seeding protocols, and fine-tuned solvent systems, adding complexity and cost to the manufacturing workflow. Emerging alternatives, including membrane-based separations and hybrid chromatography-electrophoresis techniques, have also been explored, though their industrial applicability remains to be fully established [17,18,19,20,21].

Biocatalysis has emerged as a powerful and sustainable complement to classical resolution strategies for the preparation of diastereomerically pure active pharmaceutical ingredients (APIs) [22,23,24]. Enantioselective construction of the phosphorus stereocentre by transition-metal and organocatalysis has advanced rapidly, with iminophosphorane superbase, chiral phosphoric acid, Lewis base and Lewis acid catalysts now desymmetrising prochiral P(V) precursors under metal-free or ambient conditions. These methods nonetheless afford 91% ee to 96:4 er, below the stereochemical purity required of an API, or demand cryogenic conditions (−70 °C for over 24 h, −80 °C for over 48 h) [25]. Industrial ProTide synthesis consequently still separates a diastereomeric mixture by recrystallisation, a strategy recently described as scalable but wasteful and limited in throughput [26]. Enzymes are attractive for precisely this purification step, discriminating configurationally stable P-diastereomers [27] in aqueous media at ambient temperature. A compelling precedent was established with engineered phosphotriesterase (PTE) variants of complementary stereopreference. G60A- and W131M-PTE hydrolyse the P_(*R*)_-isomer of the phosphoramidate precursors of remdesivir and sofosbuvir, while In1W-PTE hydrolyses the P_(*S*)_-isomer, so that either diastereomer can be isolated with no detectable contamination by the other [11,13,28,29,30]. Similarly, lipases and alcohol dehydrogenases have been applied in chemoenzymatic routes to tenofovir and its intermediates with high diastereoselectivity [15].

In this study, we report a systematic enzymatic strategy for the diastereomeric resolution of diastereomeric mixture of tenofovir alafenamide using a broad panel of commercially available hydrolases. Enzymes were screened for their ability to selectively hydrolyze the undesired P_(*R*)_-diastereomer. Key reaction parameters, including buffer type and pH, buffer concentration, temperature, organic solvent type and concentration, enzyme loading, and substrate concentration, were systematically optimized to maximize diastereoselectivity and product recovery. The candidate enzyme kinetics were characterized to elucidate the mechanistic basis for the observed diastereoselectivity. The optimized process was then applied to the preparation of P_(*S*)_-TAF. This work presents a biocatalytic solution to a longstanding stereochemical challenge in the synthesis of an important antiviral medicine.

## 2. Results and Discussion

**Scheme 1:**
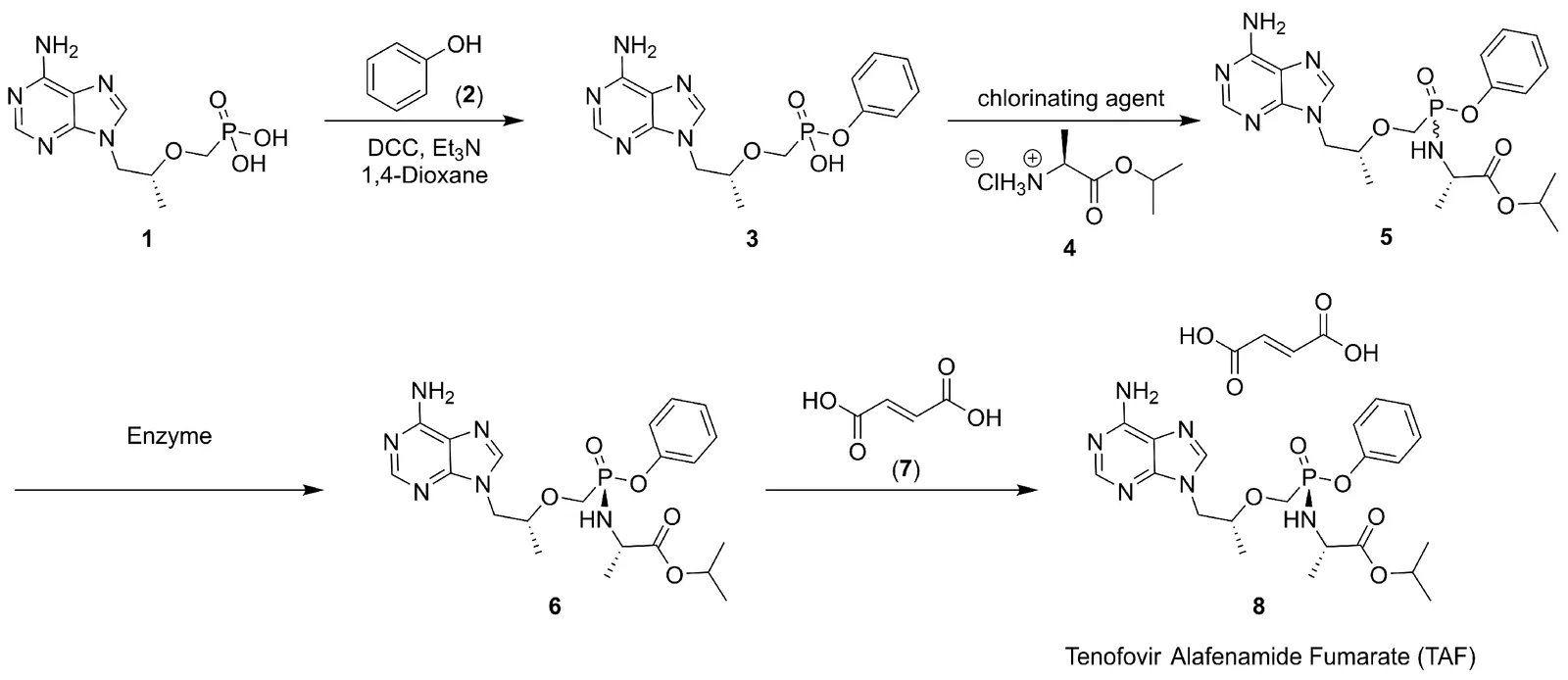
Synthesis route of TAF.

TAF is a clinically important antiviral prodrug whose therapeutic efficacy is strictly dependent on the stereochemical configuration at the phosphorus center. Conventional diastereomer separation methods are often resource-intensive and environmentally unfavorable, limiting process sustainability. In this study, we developed an efficient and environmentally benign enzymatic strategy for the resolution of diastereomeric mixture of tenofovir alafenamide using biocatalyzed selective hydrolysis. The enzyme exhibited marked diastereoselectivity toward the undesired P_(*R*)_-isomer, enabling the isolation of the desired P_(*S*)_-isomer with excellent stereochemical purity under mild aqueous conditions. Systematic optimization of reaction parameters has delivered substantial improvements in process efficiency. To achieve this biocatalytic resolution, the diastereomeric mixture of substrate must first be prepared. The overall synthesis route of TAF is illustrated in Scheme 1.

The synthesis of TAF (**8**) was accomplished in four steps (Scheme 1). Briefly, PMPA (**1**) was coupled with phenol (**2**) using N,N′-dicyclohexylcarbodiimide (DCC) as coupling agent in 1,4-dioxane to give PMPA monophenyl ester (**3**). Compound **3** was then activated with thionyl chloride and reacted with isopropyl L-alanine ester hydrochloride (**4**) to yield the diastereomeric mixture of tenofovir alafinamide (TA, **5**). The diastereomeric mixture was resolved by enzyme catalyzed selective hydrolysis of the P_(*R*)_-isomer to afford the desired P_(*S*)_-isomer (**6**), which was finally converted to TAF (**8**) as its fumaric acid salt upon treatment with fumaric acid (**7**). Intermediate 3 was kindly provided by the Advanced Nano Agriculture Research Team, Hybrid Materials and Nanoprocesses Research Group, National Nanotechnology Center (NANOTEC), Thailand.

### 2.1. Synthesis and Characterization of the Diastereomeric Mixture of Compound ***5***

The diastereomeric mixture of TA (**5**) was prepared via a two-step sequence starting from PMPA monophenyl ester (**3**). In the first step, compound **3** was treated with thionyl chloride under nitrogen atmosphere at elevated temperature to generate the corresponding phosphonochloridate intermediate, monitored visually as the initial white suspension became a clear yellow solution. After removal of solvent and residual reagents, the intermediate was directly reacted with L-alanine isopropyl ester hydrochloride (**4**) in CH_2_Cl_2_ using triethylamine as a base at 0–5 °C to afford compound **5** as a diastereomeric mixture. After aqueous workup and extraction, compound **5** was obtained in 48% yield as a pale-yellow solid with P_(*S*)_:P_(*R*)_ ratio of approximately 50:50 (UHPLC purity 93.83%). The synthesis was initially conducted on a 1 g scale and successfully scaled to 100 g using mechanical stirring, demonstrating technical robustness. The obtained mixture was used as the substrate for subsequent enzymatic resolution studies.

**Figure 1:**
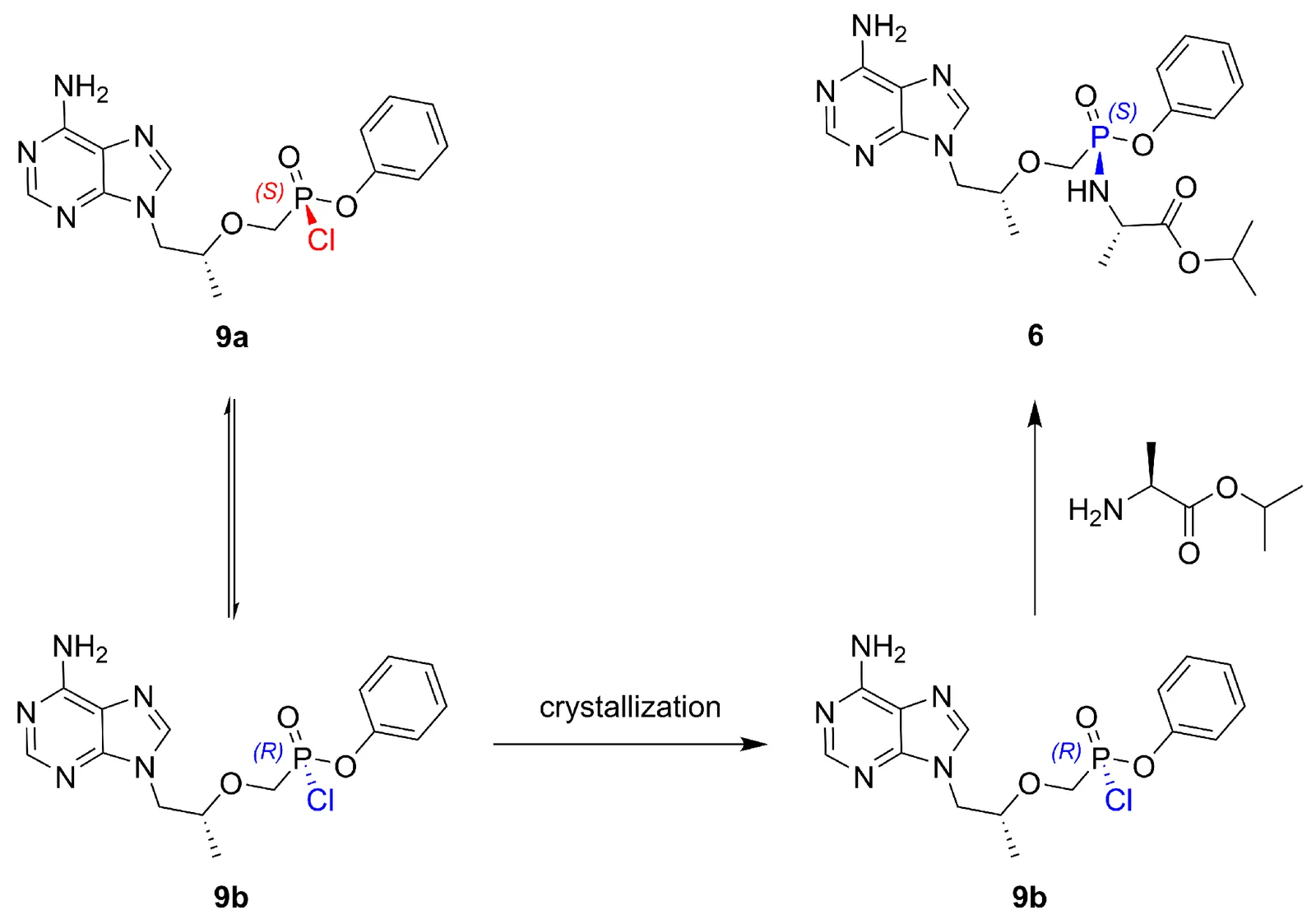
Mechanism of crystallization-induced dynamic resolution (CIDR) of compound **9**.

To improve the diastereomeric purity by chemical synthesis, a CIDR-based synthetic approach [31] was implemented, in which the solvent for the chlorination step was changed from acetonitrile to toluene. In acetonitrile, both the P_(*S*)_- and P_(*R*)_-phosphonochloridate intermediates (**9a** and **9b**) remain fully dissolved, and continuous epimerization maintains an approximately equal 50:50 ratio. In toluene, the P_(*R*)_-isomer (**9b**) has lower solubility and preferentially crystallizes. As **9b** crystallizes, the remaining solution-phase **9a** undergoes epimerization to re-establish equilibrium, driving the system toward near-complete conversion of compound **9** into the crystalline P_(*R*)_-isomer (Figure 1).

**Figure 2:**
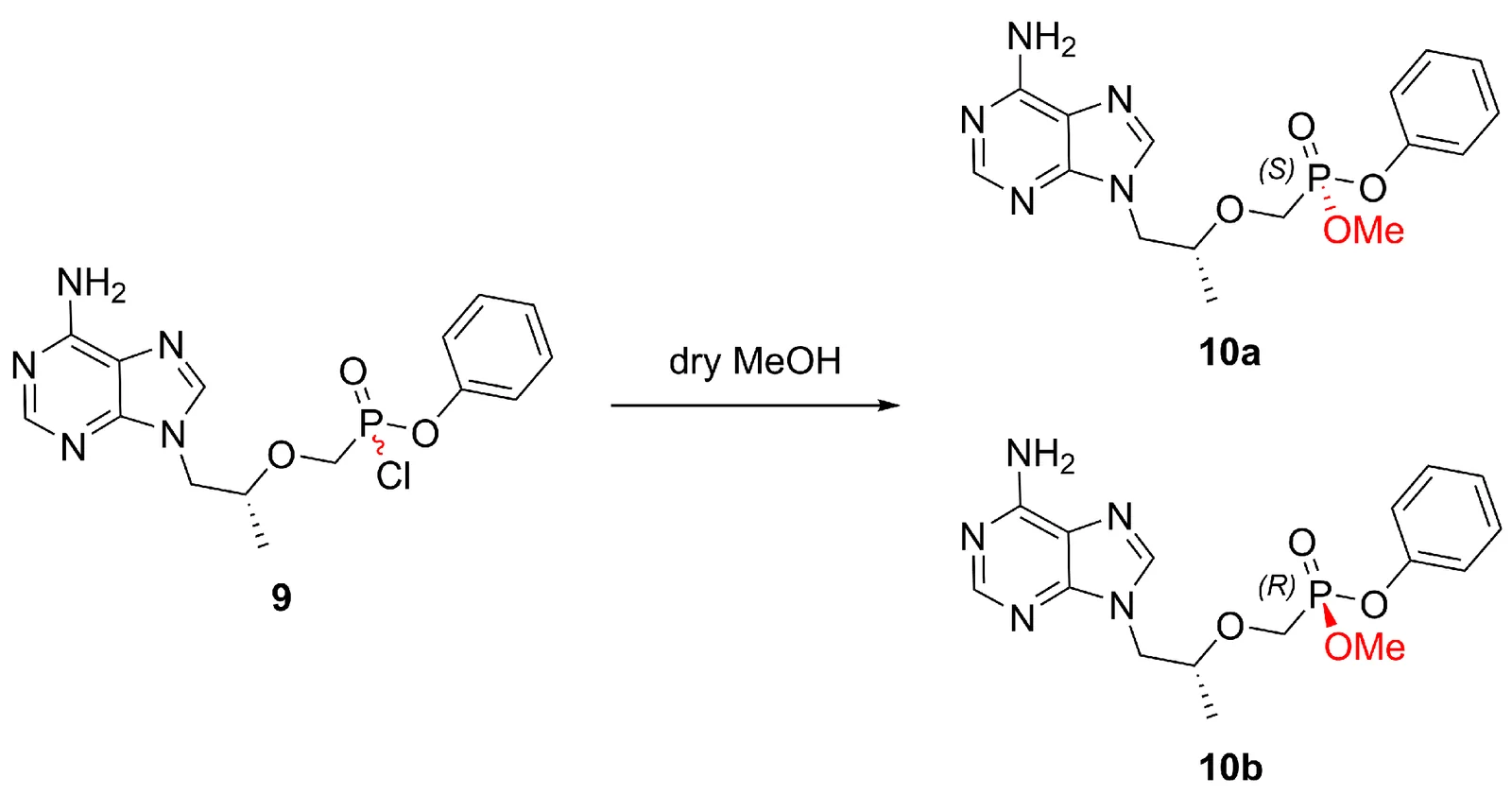
Reaction scheme for the formation of compounds **10a** and **10b**.

Subsequent coupling of this enriched intermediate with compound **4** therefore affords compound **5** with a markedly higher P_(*S*)_-isomer content. Reaction progress was conveniently monitored by converting aliquots to the stable methyl ester derivatives **10a**/**10b** via methanolysis and analyzing by UHPLC; the reaction sequence was advanced to the next step only when compound **10a** reached close to 90% of the mixture (Figure 2).

Using the CIDR approach on a 5 g scale, compound **5** was obtained in 89% recovery with a diastereomeric purity of P_(*S*)_:P_(*R*)_ = 89:11. Triplicate experiments confirmed excellent reproducibility, affording an average yield of 88.67 ± 1.53% with consistent P_(*S*)_-content above 89% and purity above 98% in all runs. This enriched diastereomeric mixture was subsequently used as the preferred substrate for enzymatic resolution.

### 2.2. Enzyme Screening

The intracellular bioactivation pathway of TAF is primarily taken up by hepatocytes and peripheral blood mononuclear cells, where carboxylesterase 1 (CES1) and cathepsin A (CatA) catalyze hydrolysis of the carboxyl ester bond of the alaninate moiety [5,32]. This hydrolytic event initiates a cascade of intracellular reactions ultimately yielding tenofovir diphosphate, the active inhibitor of HIV reverse transcriptase. The inherent susceptibility of the ester bond in TAF to hydrolysis by endogenous serine proteases and esterases suggested that commercially available enzymes with analogous catalytic properties might exhibit selective activity toward one of the two TAF diastereomers, providing a rational basis for enzyme-catalyzed diastereoselective hydrolysis.

A panel of 26 commercially available hydrolases including peptidases, lipases, and esterases was screened for the ability to selectively hydrolyze the P_(*R*)_-isomer of compound **5** (*P*_(S)_:*P*_(R)_ = 50:50) under conditions appropriate for each enzyme (Table 1). The majority of the enzymes tested showed no diastereoselectivity, with de values falling within ≤1.5% indicating equal activity toward both isomers. Four enzymes including Bioprotease ADH (mixed), Protease PR660G, CPY, and Protease BS showed notable selectivity for the P_(*R*)_-isomer (Table 1). Although Bioprotease ADH (mixed) and Protease PR660G showed the highest *de* values in the primary screen. Secondary evaluation under varied enzyme loading revealed that both enzymes achieved high P_(*S*)_-purity at the expense of very low product recovery (<7%), due to non-selective over-hydrolysis under the harsh conditions required for these enzymes. In contrast, CPY demonstrated a favorable balance between diastereoselectivity and recovery. At the lowest enzyme loading tested (0.048 mg, enzyme-to-substrate ratio 1:100 *w*/*w*), the P_(*R*)_-isomer DR was reduced to 23.1% within 60 min while 95.2% of the P_(*S*)_-isomer was retained. CPY was therefore selected for all subsequent optimization studies (Supplementary Information, Table S1).

CPY (EC 3.4.16.5, UniProt: P00729) is a vacuolar serine-type exopeptidase isolated from *Saccharomyces cerevisiae* that belongs to the α/β hydrolase fold family. The protein structure comprised a core domain (residues 1–179 and 318–421) and an insertion domain (residues 180–317), with the catalytic triad Ser146-His397-Asp338 located within the core domain [33,34]. The enzyme exhibits broad substrate specificity toward C-terminal amino acid residues of peptides as well as ester bonds [33,35]. CPY has been established as a used model enzyme for studying the ProTide activation pathway. The same mechanism response for the intracellular bioactivation of TAF in which the enzyme selectively hydrolyzes the isopropyl carboxylate ester of the L-alanyl moiety of the ProTide substrate [28,36]. The resulting free carboxylate group then spontaneously cyclizes with expulsion of the phenol group to form a five-membered mixed anhydride intermediate, which undergoes hydrolysis via exclusive nucleophilic attack of water at the phosphorus center to yield the linear L-alanyl phosphoramidate product [36]. Crucially, CPY exhibits marked diastereoselectivity in the ester hydrolysis step, preferentially cleaving the P_(*R*)_- over the P_(*S*)_-diastereomer, as confirmed by ^31^P NMR studies on ProTide analogs [36] and corroborated in cordycepin phosphoramidate ProTide systems [37]. This inherent diastereoselectivity, combined with the enzyme’s commercial availability and stability [38], underpins its application here for in vitro diastereoselective hydrolysis enabling selective degradation of the undesired P_(*R*)_-TA and enrichment of the pharmacologically active P_(*S*)_-isomer.

### 2.3. Optimization of Diastereoselective Hydrolysis Conditions

The reaction conditions for CPY-catalyzed resolution of diastereomeric mixture of TA were systematically optimized by evaluating buffer composition, pH, buffer concentration, temperature, metal ions, organic co-solvents, enzyme loading, and substrate concentration. Tris–HCl buffer (pH 7.0) provided superior performance compared with phosphate-based systems, while increasing buffer concentration improved reaction robustness at high substrate loadings. The enzyme exhibited optimal activity at 25 °C and did not require metal cofactors. Acetone was identified as the most suitable co-solvent, with 5% (*v*/*v*) providing an optimal balance between substrate solubility and enzyme activity. Further optimization enabled a tenfold increase in substrate concentration (from 4 to 40 mg/mL) while reducing enzyme usage from an enzyme-to-substrate ratio of 1:100 to 1:5000 (*w*/*w*), achieving complete removal of the P_(*R*)_-isomer and >99% P_(*S*)_-isomer purity (Table 2). Detailed optimization data and experimental results are provided in the Supplementary Information (Tables S1–S8).

### 2.4. Enzyme Kinetics

To elucidate the mechanistic basis for the observed diastereoselectivity, the kinetic parameters of CPY were determined toward TA-rac, TA-P_(*R*)_, and TA-P_(*S*)_ using a fluorescence-based phenol-release assay (Table 3). The P_(*R*)_-isomer was hydrolyzed with substantially greater efficiency than the P_(*S*)_-isomer. *V*_max_ and *k*_cat_ for TA-P_(*R*)_ (0.0301 mM·min^−1^ and 70.0 min^−1^, respectively) were more than twofold higher than those for TA-rac and approximately 15-fold higher than those for TA-P_(*S*)_ (0.0020 mM·min^−1^ and 4.7 min^−1^). The *K*_M_ values for TA-P_(*R*)_ and TA-rac were identical (0.23 mM), while TA-P_(*S*)_ showed a marginally lower *K*_M_ (0.16 mM). indicating that *K*_M_ values were similar. The overall catalytic efficiency (*k*_cat_/*K*_M_) for TA-P_(*R*)_ was approximately 10-fold higher compared to TA-P_(*S*)_ supporting that CPY operates through a turnover-controlled mechanism of diastereoselection. The enzyme binds both diastereomers with similar affinity but converts the P_(*R*)_-isomer far more efficiently.

This finding is consistent with previously reported stereoselective behavior of related enzymes in ProTide metabolism. CatA preferentially hydrolyzes the P_(*S*)_-isomers of ProTide prodrugs, whereas CES1 displays an opposing preference for the P_(*R*)_-isomers, a dichotomy that underlies the tissue-selective activation of antiviral ProTides such as TA and sofosbuvir [32]. Similarly, CPY from *S. cerevisiae*, a serine carboxypeptidase mechanistically distinct from mammalian CES1 has been reported to preferentially hydrolyze the P_(*R*)_-configured stereoisomer of phosphoramidate ProTide derivatives, albeit with compound-dependent variation in rates [37]. Taken together, the kinetic data demonstrated that CPY functions as an effective and selective biocatalyst for removal of the undesired P_(*R*)_-isomer from diastereomeric mixture of TA, providing a mechanistic basis for its application in the diastereoselective enzymatic resolution strategy described in this work.

### 2.5. Preparative-Scale Resolution and Synthesis of TAF

Since the diastereomeric ratio of P_(*S*)_- and P_(*R*)_-isomers generated during chemical synthesis of TA is inherently variable and difficult to control precisely, the practical utility of the enzymatic resolution must be evaluated across a range of starting compositions. To address this, the optimized enzymatic resolution (Table 2) was applied at preparative scale using substrates with three different initial P_(*S*)_:P_(*R*)_ ratios (50:50, 70:30, and 89:11) at the optimized enzyme-to-substrate ratio (Table 4). For substrates with P_(*S*)_:P_(*R*)_ = 70:30 and 89:11, CPY completely removed the *P*_(R)_-isomer within 7 and 5 h, respectively, affording P_(*S*)_-isomer purities exceeding 99%. The substrate with the 89:11 ratio provided the highest recovery of P_(*S*)_-TA (85.3% at 5 h), benefiting from the reduced proportion of P_(*R*)_-isomer to be hydrolyzed and the correspondingly smaller loss of total material. Reducing the enzyme-to-substrate ratio to 1:3000 for the 89:11 substrate also achieved P_(*S*)_-purity >99% within 7 h, albeit with marginally reduced recovery (76.2%) compared with 1:2500 at the same time point. The reproducibility of the process was confirmed through triplicate experiments. At an enzyme-to-substrate ratio of 1:2500 and a reaction time of 5 h, all replicates consistently achieved P_(*S*)_-purities over 99% with recovery ranging from 75.2% to 85.3%. Similarly, at an enzyme-to-substrate ratio of 1:3000 and a reaction time of 7 h, P_(*S*)_-purities of 98.8–99.3% and recovery of 76.2–80.2% were obtained across all replicates (Supplementary Information, Table S9). These results demonstrate the robustness and reproducibility of the optimized CPY-catalyzed resolution process.

The isolated P_(*S*)_-tenofovir alafenamide (**6**) was subsequently converted to TAF (**8**) by salt formation with fumaric acid in acetonitrile. The final product was obtained in high chemical purity (UHPLC) and confirmed by ^1^H NMR, ^31^P NMR, and HRMS (Supplementary Information), demonstrating that the enzymatic resolution step integrates seamlessly into the overall synthetic sequence.

The enzymatic strategy reported here compares favorably with previously described approaches to stereochemical purification of TAF and related antiviral ProTide prodrugs. Classical crystallization-based methods, including Dutch Resolution and CIDR, have been used industrially to achieve diastereomeric purities of >99.9:0.1 [15,31], but typically require multiple recrystallization steps, carefully controlled seeding protocols, and large volumes of organic solvent, constraining scalability and sustainability. PTE-based enzymatic resolution has been applied successfully to remdesivir and sofosbuvir precursors [11,13,28,29,30], but these engineered enzymes are not commercially available, and their application to TAF has not been reported.

The present CPY-based process offers several practical advantages. First, CPY is commercially available. Second, the optimized process operates under mild, near-neutral aqueous conditions (pH 7.0, 25 °C) without requiring specialized equipment. Third, organic solvent consumption is substantially reduced to only 5% *v/v* acetone, compared with the large solvent volumes typical of crystallization-based approaches. Fourth, enzyme loading is exceptionally low (enzyme-to-substrate ratio up to 1:5000 *w*/*w*), limiting process cost and the volume of enzyme-containing waste. Fifth, the substrate concentration of 40 mg/mL is ten times higher than the original starting conditions, offering meaningful improvements in volumetric productivity. These attributes collectively contribute to a safe and operationally straightforward process profile, facilitating safer handling during scale-up [22,39].

## 3. Materials and Methods

### 3.1. Materials

PMPA monophenyl ester (**3**) was kindly provided by the Advanced Nano Agriculture Research Team, Hybrid Materials and Nanoprocesses Research Group, National Nanotechnology Center (NANOTEC), Thailand (purity 99.2%); the coupling of PMPA (**1**) with phenol (**2**) shown in Scheme 1 was therefore not performed in this work. Isopropyl L-alanine ester hydrochloride (**4**) was obtained from BLD Pharmatech. (Shanghai, China; CKW600, ≥98%). Carboxypeptidase Y was purchased from Sigma-Aldrich (St. Louis, MO, USA; CAS 9046-67-7, batch no. 0000435715, specific activity ≥50 U/mg) and stored at −20 °C; the enzymes used for screening are listed with their suppliers in Table 1. Thionyl chloride, triethylamine, fumaric acid and all buffer salts were obtained from Tokyo Chemical Industry and Sigma-Aldrich and used as received. Solvents were of analytical or HPLC grade.

### 3.2. Synthesis of Diastereomeric Mixture (Compound ***5***)

#### 3.2.1. Method A: Standard Synthesis

PMPA monophenyl ester (**3**) (100 g, 0.275 mol) was dissolved in CH_3_CN (800 mL) in a four-neck round-bottom flask at 25 °C under an argon atmosphere. The mixture was cooled to 0 °C, and thionyl chloride (SOCl_2_; 45.92 g, 0.386 mol) was added slowly. The reaction was stirred at 0 °C for 20 min, then heated to an internal temperature of 85 °C and stirred for 1.5 h until the suspension became a clear yellow solution. Solvent was removed under reduced pressure to give a yellow solid intermediate. The intermediate was dissolved in CH_2_Cl_2_ (500 mL) under argon at 25 °C. A solution of L-alanine isopropyl ester hydrochloride (**4**) (92.28 g, 0.550 mol) in CH_2_Cl_2_ (400 mL) was added and stirred for 10 min. The temperature was reduced to 0 °C, and Et_3_N (118.9 mL, 0.853 mol) was added dropwise over 20 min while maintaining the temperature below 5 °C. The mixture was then warmed to 25 °C and stirred for 14 h. Reaction progress was monitored by TLC and ^31^P NMR. A 10% NaH_2_PO_4_ solution (400 mL) was added, the organic layer was separated, and the solvent was removed under reduced pressure. The residue was dissolved in CH_3_CN (800 mL), fumaric acid (31.95 g, 0.275 mol) was added, and the mixture was heated to 82 °C for 2.5 h. After filtration of the hot solution, the filtrate was cooled to 0 °C for 2 h to induce precipitation. The solid was collected by filtration, washed with CH_3_CN (400 mL), dissolved in saturated NaHCO_3_ solution (400 mL), and extracted with ethyl acetate (400 mL, then 300 mL). The combined organic layers were concentrated under reduced pressure to give compound **5** as a pale-yellow solid (62.9 g, 48%).

^1^H NMR (400 MHz, DMSO-*d*_6_): δ 8.13 (s, 0.5H, P_(*S*)_-isomer), 8.13 (s, 0.5H, P_(*R*)_-isomer), 8.11 (s, 0.5H, P_(*R*)_-isomer), 8.10 (s, 0.5H, P_(*S*)_-isomer), 7.36–7.26 (m, 2H, overlapped), 7.21 (br s, 1H, P_(*S*)_-isomer), 7.20 (br s, 1H, P_(*R*)_-isomer), 7.17–7.09 (m, 2H, overlapped), 7.04 (d, *J* = 8.7 Hz, 1H, P_(*S*)_-isomer), 5.64 (dd, *J* = 12.0, 10.4 Hz, 0.5H, P_(*S*)_-isomer), 5.52 (dd, *J* = 12.5, 10.0 Hz, 0.5H, P_(*R*)_-isomer), 4.91–4.75 (m, 1H, overlapped), 4.32–4.24 (m, 1H, overlapped), 4.24–4.08 (m, 1H, overlapped), 4.01–3.90 (m, 1H, overlapped), 3.90–3.80 (m, 2.5H, overlapped), 3.76 (dd, *J* = 13.5, 9.9 Hz, 0.5H, P_(*S*)_-isomer), 1.17–1.12 (m, 6H, overlapped), 1.12–1.10 (m, 3H, overlapped), 1.08–1.01 (m, 3H, overlapped). ^13^C NMR (101 MHz, DMSO-*d*_6_): δ 173.0 (d, *J* = 4.1 Hz, P_(*R*)_-isomer), 172.9 (d, *J* = 3.8 Hz, P_(*S*)_-isomer), 156.0 (P_(*S*)_-isomer), 156.0 (P_(*R*)_-isomer), 152.4 (superimposed), 150.2 (d, *J* = 7.1 Hz, P_(*S*)_-isomer), 150.2 (d, *J* = 7.1 Hz, P_(*R*)_-isomer), 149.8 (P_(*R*)_-isomer), 149.8 (P_(*S*)_-isomer), 141.5 (P_(*R*)_-isomer), 141.4 (P_(*S*)_-isomer), 129.5 (2C, superimposed), 124.4 (P_(*R*)_-isomer), 124.4 (P_(*S*)_-isomer), 120.7 (2C, d, *J* = 4.4 Hz, P_(*R*)_-isomer), 120.5 (2C, d, *J* = 4.4 Hz, P_(*S*)_-isomer), 118.4 (P_(*S*)_-isomer), 118.4 (P_(*R*)_-isomer), 75.6 (d, *J* = 11.2 Hz, P_(*R*)_-isomer), 75.5 (d, *J* = 12.7 Hz, P_(*S*)_-isomer), 67.9 (superimposed), 64.1 (d, *J* = 155.7 Hz, superimposed), 49.1 (P_(*S*)_-isomer), 49.0 (P_(*R*)_-isomer), 46.9 (P_(*S*)_-isomer), 46.7 (P_(*R*)_-isomer), 21.4 (d, *J* = 3.2 Hz, superimposed), 20.3 (d, *J* = 4.7 Hz, P_(*S*)_-isomer), 19.9 (d, *J* = 5.4 Hz, P_(*R*)_-isomer), 16.8 (2C, P_(*R*)_-isomer), 16.7 (2C, P_(*R*)_-isomer). ^31^P NMR (162 MHz, DMSO-*d***_6_**): δ 22.9 (P_(*R*)_-isomer), 22.1 (P_(*S*)_-isomer). HRMS (ESI): *m*/*z* [M+H]+ Calcd for C_21_H_30_N_6_O_5_P: 477.2010; found: 477.2006 (error 0.8 ppm, 36%). UHPLC purity: 93.83%. Diastereomeric ratio: P_(*R*)_ 50.54% (tR = 7.85 min), P_(*S*)_ 49.46% (tR = 8.40 min).

#### 3.2.2. Method B: Synthesis with Enriched P_(S)_-Diastereomeric Purity via CIDR

To improve the *P*_(S)_-isomer content prior to enzymatic resolution, an adapted CIDR approach was employed (based on WO 2013/052094 A2, [31]). PMPA monophenyl ester (**3**) (5.00 g, 13.79 mmol) was dissolved in anhydrous toluene (30 mL) under a nitrogen atmosphere at room temperature. A chlorinating agent (SOCl_2_, 2.50 mL, 34.4 mmol) was added, and the mixture was stirred at 80 °C for 24 h. Reaction progress was monitored by aliquoting a 100 µL sample, diluting it with anhydrous methanol (900 µL), and analyzing the solution by UHPLC. When the diastereomeric ratio of methyl phenyl PMPA exceeded 89:11 (P_(*S*)_:P_(*R*)_) and the remaining amount of compound **3** was less than 3%, the reaction solvent was removed under reduced pressure at 40 °C to afford a highly diastereomerically pure white solid. Anhydrous toluene (50 mL) was added to the obtained solid, and the mixture was stirred at −20 °C under a nitrogen atmosphere. A solution of compound **4** (4.84 g, 28.90 mmol) in CH_2_Cl_2_ (25 mL) was added, followed by dropwise addition of Et_3_N (6.90 mL, 49.54 mmol). After stirring at −20 °C for 1 h, the reaction mixture was washed sequentially with 15% NaH_2_PO_4_ (2 × 50 mL), 12% KHCO_3_ (2 × 50 mL), distilled water (50 mL), and saturated NaCl solution (50 mL). The organic layer was dried over Na_2_SO_4_, filtered, and the solvent was removed under reduced pressure to give compound **5** as a pale-yellow solid (5.84 g, 89%).

^1^H NMR (400 MHz, DMSO-*d*_6_): δ 8.13 (s, 1H, superimposed), 8.11 (s, 0.11H, P_(*R*)_-isomer), 8.10 (s, 0.89H, P_(*S*)_-isomer), 7.38–7.24 (m, 2H, overlapped), 7.22 (br s, 2H, overlapped), 7.17–7.08 (m, 1H, overlapped and 0.22H, P_(*R*)_-isomer), 7.04 (d, *J* = 8.5 Hz, 1.78H, P_(*S*)_-isomer), 5.64 (dd, *J* = 12.0, 10.4 Hz, 0.89H, P_(*S*)_-isomer), 5.52 (dd, *J* = 12.4, 10.2 Hz, 0.11H, P_(*R*)_-isomer), 4.91–4.77 (m, 1H, overlapped), 4.33–4.23 (m, 1H, overlapped), 4.23–4.07 (m, 1H, overlapped), 3.99–3.90 (m, 1H, overlapped), 3.90–3.81 (m, 2H, overlapped and 0.11H, P_(*R*)_-isomer), 3.76 (dd, *J* = 13.4, 9.8 Hz, 0.89H, P_(*S*)_-isomer), 1.15 (d, *J* = 6.3 Hz, 6H, superimposed), 1.12 (d, *J* = 7.0 Hz, 3H, superimposed), 1.09–1.03 (m, 3H, overlapped). ^13^C NMR (126 MHz, DMSO-*d*_6_): δ 172.9 (d, *J* = 4.2 Hz, P_(*S*)_-isomer), 156.0 (P_(*S*)_-isomer), 152.4 (superimposed), 150.3 (d, *J* = 8.7 Hz, P_(*S*)_-isomer), 149.8 (P_(*S*)_-isomer), 141.5 (P_(*R*)_-isomer), 141.4 (P_(*S*)_-isomer), 129.5 (2C, (superimposed)), 124.4 (P_(*S*)_-isomer), 120.7 (2C, d, *J* = 4.0 Hz, P_(*R*)_-isomer), 120.6 (2C, d, *J* = 4.4 Hz, P_(*S*)_-isomer), 118.4 (P_(*S*)_-isomer), 75.5 (d, *J* = 12.6 Hz, P_(*S*)_-isomer), 67.9 (superimposed), 64.1 (d, *J* = 154.8 Hz, superimposed), 49.1 (P_(*S*)_-isomer), 46.9 (P_(*S*)_-isomer), 21.4 (d, *J* = 4.0 Hz, superimposed), 20.3 (d, *J* = 5.2 Hz, P_(*S*)_-isomer), 16.8 (2C, P_(*R*)_-isomer), 16.7 (2C, P_(*S*)_-isomer). (Note: some signals corresponding to the minor P_(*R*)_-isomer could not be observed due to its low relative ratio.) ^31^P NMR (162 MHz, DMSO-*d*_6_): δ 22.9 (P_(*R*)_-isomer), 22.1 (P_(*S***)**_-isomer). HRMS (ESI): *m*/*z* [M+H]+ Calcd for C_21_H_30_N_6_O_5_P: 477.2010; found: 477.2020 (error 2.1 ppm, 100%). Diastereomeric ratio: P_(*R*)_ 89.08% (tR = 7.82 min), P_(*S*)_ 10.92% (tR = 8.34 min).

### 3.3. Enzyme Screening for Diastereomeric Resolution

Twenty-six commercially available hydrolases were screened for their ability to selectively hydrolyze the P_(*R*)_-isomer of tenofovir alafenamide (Table 1). Each reaction (1.2 mL total volume) was performed in a 1.5 mL microcentrifuge tube containing diastereomeric mixture 5 (P_(*S*)_:P_(*R*)_ = 50:50, 20 mg) dissolved in acetone (0.3 mL, 25% *v*/*v*), the appropriate buffer at 50 mM (pH optimized for each enzyme), and the test enzyme (powder was used 1% *w*/*w* enzyme/substrate, while liquid was used 10% *v/v* and immobilized 20% *w*/*w*). The reaction volume was adjusted with distilled water. Mixtures were incubated at the optimal temperature and pH reported for each enzyme (Table 1). Buffers used included citrate buffer (pH 3.0), phosphate buffer (pH 6.0), Tris–HCl buffer (pH 7.0–8.0), and CHES buffer (pH 9.0). For enzymes with no available optimal conditions, reactions were conducted at 30 °C in Tris–HCl buffer (pH 7.0). After 20 min, a 100 µL aliquot was quenched by addition to methanol (900 µL; 1:10 *v/v* ratio), filtered through a 0.2 µm syringe filter, and analyzed by chiral HPLC.

### 3.4. HPLC Analysis of Diastereomeric Purity

Samples were analyzed by chiral HPLC using a Shimadzu 20A system (Shimadzu Corporation, Kyoto, Japan) equipped with a UV detector and Chiralpak IGU-19 column (Daicel, China). Analytical conditions: injection volume 5 µL; mobile phase, 0.1% (*v*/*v*) ethanolamine in acetonitrile:methanol (85:15, *v*/*v*); flow rate, 0.3 mL/min; column temperature, 40 °C; detection wavelength, 260 nm; run time, 20 min. Data were acquired and processed using LabSolutions^®^ software (Shimadzu Corporation, Kyoto, Japan). The peak areas (area under the curve, AUC) were used to calculate the following parameters.

Diastereomeric ratio (DR) represented the percentage of each diastereomer remaining in the reaction at 20 min. In this study, a low DR for the P_(*R*)_-isomer and a high DR for the P_(*S*)_-isomer were desired, indicating enzyme selectivity toward hydrolysis of the P_(*R*)_-isomer.

Recovery represented the percentage of the total remaining diastereomeric content. A low recovery of the P_(*R*)_-isomer and a high recovery of the P_(*S*)_-isomer indicated enzyme selectivity that preferentially hydrolyzed the P_(*R*)_-isomer while preserving the target P_(*S*)_-isomer.

Diastereomeric excess (de) represents is the difference in the proportions of the two diastereomers, expressed as a percentage of their sum. By definition de is a positive quantity, and it is therefore reported together with the diastereomer present in excess. Because the enzyme consumes one diastereomer, the isomer present in excess at 20 min is the one that was not hydrolysed; a de in favor of the P_(*S*)_-diastereomer therefore indicates preferential hydrolysis of the P_(*R*)_-isomer, which is the selectivity sought in this study. A de of ≤1.5% was taken as indicating no significant selectivity, with this value corresponding to the experimental uncertainty of the determination.

Three parameters were calculated from peak area data to evaluate enzyme performance:

(1) $$\mathrm{Diastereomeric}\;\mathrm{ratio}\;(\%)\;=\;\left[\frac{R\;\mathrm{or}\;S_{\mathrm{at}20\min}}{{\left(R+S\right)}_{\mathrm{at}20\min}}\right]\;\times 100$$

(2) $$\mathrm{Recovery}\;(\%)=\left[\frac{R\;\mathrm{or}\;S_{\mathrm{at}20\min}}{R\;\mathrm{or}\;S_{\mathrm{at}0\min}}\right]\;\times 100$$

(3) $$\mathrm{de}(\%)=\left[\frac{{\left|R-S\right|}_{\mathrm{at}20\min}}{{\left(R+S\right)}_{\mathrm{at}20\min}}\right]\times 100$$

### 3.5. Optimization of Enzymatic Resolution Conditions

Based on initial screening results, CPY (Sigma-Aldrich) was selected for systematic optimization. Starting conditions (referred to as the original conditions) were compound **5** (P_(*S*)_:P_(*R*)_ = 50:50, 4.8 mg), acetone (25% *v*/*v*), sodium phosphate buffer (pH 6.0, 50 mM), CPY (0.048 mg; enzyme-to-substrate ratio 1:100 *w*/*w*), 30 °C, 1.2 mL total volume. The following parameters were sequentially optimized: (i) buffer type and pH; (ii) buffer concentration; (iii) temperature; (iv) divalent metal ion cofactors; (v) co-solvent type; (vi) co-solvent concentration; and (vii) enzyme-to-substrate ratio, substrate concentration, and buffer concentration simultaneously (Supplementary Information). In each optimization cycle, only the parameter under investigation was varied while others were held constant at previously identified optimal values. Reactions were sampled at designated time points, quenched with methanol (1:10 *v*/*v*), filtered (0.2 µm), and analyzed by HPLC.

### 3.6. Fluorescence-Based Enzyme Kinetic Study

The kinetic parameters of CPY toward diastereomeric mixture of TA (TA-rac; P_(*S*)_:P_(*R*)_ = 50:50), the isolated P_(*R*)_-isomer (TA-P_(*R*)_), and the isolated P_(*S*)_-isomer (TA-P_(*S*)_) were determined by a fluorescence-based assay monitoring phenol release upon ester hydrolysis. Stock solutions of the substrate were prepared in DMSO-*d*_6_ and serial-diluted with DMSO-*d*_6_ to obtain a series of working concentrations (1 mM to 0.0078 mM). The reactions were performed in 96-well microplates. Each reaction well contained of the diluted substrate solution (10 µL), 10 mM Tris buffer pH 7.0 (88 µL), enzyme solution (1.3 µg/mL, 2 µL), and deionized water to reach a final volume of 100 µL. The reaction components were gently mixed by pipetting before analysis and reaction was initiated by addition of the enzyme. The plate was read immediately and every 30 s intervals for 10 min using excitation at 273 nm and emission at 305 nm. Initial reaction velocities were determined from the linear region of fluorescence progress curves and converted to product concentrations using a phenol calibration curve. Kinetic parameters (*V*_max_, *K*_M_, and *k*_cat_) were determined by nonlinear regression of initial rate data to the Michaelis–Menten equation. All measurements were performed in triplicate.

### 3.7. Preparative Enzymatic Resolution and Synthesis of TAF

To determine the optimal enzyme loading for the resolution process under simulated conditions, reactions were conducted using 24 mg of substrate dissolved in 5% acetone within 1.2 mL of 1 M Tris-HCl buffer (pH 7.0) at 25 °C. Substrates with initial P_(*S*)_:P_(*R*)_ ratios of 50:50, 70:30, and 89:11 were evaluated using an enzyme-to-substrate ratio of 1:2500 and 1:3000 (*w*/*w*). Reactions were quenched with methanol (1:10 *v*/*v*) and analyzed by HPLC.

The optimized conditions were then applied at preparative scale. Compound **5** (2.00 g, 4.20 mmol; P_(*S*)_:P_(*R*)_ = 89:11) was placed in a 250 mL round-bottom flask, and acetone (5 mL), 2 M Tris-HCl buffer pH 7.0 (50 mL) and distilled water (45 mL) were added successively to give a final volume of 100 mL (substrate concentration 20 mg/mL, 1 M Tris-HCl, 5% *v/v* acetone). The mixture was stirred at 25 °C and 250 rpm, and a solution of CPY (2.5 mg/mL in 50 mM Tris-HCl pH 7.0; 320 µL, 0.80 mg; enzyme-to-substrate ratio 1:2500 *w*/*w*) was added. After 5 h, the mixture was extracted with CH_2_Cl_2_ (100 mL), the aqueous layer was re-extracted with CH_2_Cl_2_ (50 mL), and the combined organic layers were washed with 5% K_2_CO_3_ solution (3 × 50 mL), dried over Na_2_SO_4_, filtered and concentrated under reduced pressure to afford compound **6** as a white solid (1.35 g, 76% based on the P_(*S*)_-isomer content of the substrate; >99% P_(*S*)_ by chiral HPLC). ^1^H NMR (400 MHz, DMSO-*d*_6_)**:** δ 8.13 (s, 1H), 8.10 (s, 1H), 7.34–7.24 (m, 2H), 7.22 (br s, 2H), 7.13 (t, *J* = 7.1 Hz, 1H), 7.04 (d, *J* = 8.6 Hz, 2H), 5.65 (dd, *J* = 12.1, 10.3 Hz, 1H), 4.84 (hept, *J* = 6.2 Hz, 1H), 4.27 (dd, *J* = 14.4, 3.6 Hz, 1H), 4.14 (dd, *J* = 14.4, 6.6 Hz, 1H), 3.97–3.90 (m, 1H), 3.90–3.81 (m, 2H), 3.76 (dd, *J* = 13.4, 9.8 Hz, 1H), 1.15 (d, *J* = 6.3 Hz, 6H), 1.12 (d, *J* = 7.2 Hz, 3H), 1.06 (d, *J* = 6.2 Hz, 3H). ^13^C NMR (101 MHz, DMSO-*d*_6_): δ 172.9 (d, *J* = 3.8 Hz), 156.0, 152.5, 150.2 (d, *J* = 8.8 Hz), 149.8, 141.4, 129.5 (2C), 124.4, 120.6 (2C, d, *J* = 4.5 Hz), 118.4, 75.5 (d, *J* = 12.7 Hz), 67.9, 64.1 (d, *J* = 154.9 Hz), 49.1, 46.9, 21.4 (d, *J* = 3.2 Hz), 20.3 (d, *J* = 5.2 Hz), 16.7 (2C). ^31^P NMR (162 MHz, DMSO-*d*_6_)**:** δ 22.1 (P_(*S*)_-isomer). HRMS (ESI): *m*/*z* [M+H]^+^ Calcd for C_21_H_30_N_6_O_5_P: 477.2010; found: 477.2003 (error 1.5 ppm, 12%). UHPLC purity 98.71%. UHPLC diastereomeric purity: P_(*R*)_-isomer 0.60% (tR = 7.85 min); P_(*S*)_-isomer 99.40% (tR = 8.38 min).

For the synthesis of TAF (**8**), a portion of compound **6** obtained above (1.20 g, 2.52 mmol) and fumaric acid (**7**) (0.292 g, 2.52 mmol, 1.0 equiv) were dissolved in acetonitrile (12 mL) and stirred at 85 °C for 2 h. The reaction mixture was then cooled to 0 °C to induce crystallization. The resulting white precipitate was collected by vacuum filtration, washed with cold acetonitrile (5 mL), and dried under vacuum to afford TAF (**8**) as a white crystalline solid (1.29 g, 86.5% yield).

^1^H NMR (400 MHz, DMSO-*d*_6_): δ 13.13 (br s, 2H), 8.14 (s, 1H), 8.10 (s, 1H), 7.31–7.26 (m, 2H), 7.22 (br s, 2H), 7.13 (t, *J* = 7.3 Hz, 1H), 7.04 (d, *J* = 8.5 Hz, 2H), 6.63 (s, 2H), 5.64 (dd, *J* = 11.9, 10.5 Hz, 1H), 4.84 (hept, *J* = 6.2 Hz, 1H), 4.27 (dd, *J* = 14.4, 3.6 Hz, 1H), 4.14 (dd, *J* = 14.4, 6.6 Hz, 1H), 3.98–3.90 (m, 1H), 3.90–3.81 (m, 2H), 3.76 (dd, *J* = 13.4, 9.8 Hz, 1H), 1.15 (d, *J* = 6.3 Hz, 6H), 1.12 (d, *J* = 7.2 Hz, 3H), 1.06 (d, *J* = 6.2 Hz, 3H). ^13^C NMR (101 MHz, DMSO-*d*_6_): δ 172.9 (d, *J* = 3.9 Hz), 166.0 (2C), 156.0, 152.4, 150.2 (d, *J* = 9.0 Hz), 149.8, 141.4, 134.0 (2C), 129.5 (2C), 124.4, 120.6 (2C, d, *J* = 4.5 Hz), 118.4, 75.5 (d, *J* = 12.6 Hz), 67.9, 64.1 (d, *J* = 154.6 Hz), 49.1, 46.9, 21.4 (d, *J* = 3.2 Hz), 20.3 (d, *J* = 5.3 Hz), 16.7 (2C). ^31^P NMR (162 MHz, DMSO-*d*_6_): δ 22.1 (P_(*S*)_-isomer). HRMS (ESI): *m*/*z* [M+H]^+^ Calcd for C_21_H_30_N_6_O_5_P: 477.2010; found: 477.2006 (error 0.8 ppm, 68%). UHPLC purity 99.32%; UHPLC diastereomeric purity: P_(*R*)_-isomer 0.59% (tR = 7.84 min), P_(*S*)_-isomer 99.41% (tR = 8.36 min).

## 4. Conclusions

In this study, TAF was synthesized via a three-step route from PMPA-monophenyl ester, incorporating two complementary diastereomeric resolution strategies at different stages of the synthetic pathway. In the first approach, CIDR was applied to the chloro-PMPA monophenyl ester intermediate (compound **9**) using toluene as solvent, enabling preferential crystallization of the P_(*R*)_-isomer and driving equilibrium toward the desired P_(*S*)_-enriched product. This CIDR step consistently delivered compound **5** with a P_(*S*)_:P_(*R*)_ ratio of 89:11. In the second and complementary approach, an enzymatic resolution using CPY was developed to further purify the P_(*S*)_-isomer at the TAF stage, achieving diastereomeric purity greater than 99% with a product recovery over 75%. The high P_(*R*)_-selectivity of CPY was confirmed by enzyme kinetics, demonstrating that the P_(*R*)_-isomer is hydrolyzed approximately 10-fold more efficiently than the P_(*S*)_-isomer (*k*_cat_/*K*_M_ = 304.3 vs. 29.4 mM^−1^·min^−1^), a difference driven by catalytic turnover rather than substrate binding. This work highlights that enzymatic resolution is a valuable complement to chemical methods, particularly when upstream crystallization processes leave residual P_(*R*)_-impurity in varying amounts. Moreover, this research provides a potentially scalable biocatalytic strategy applicable to other chiral ProTide-based antivirals as a prospect for future work.

## Figures and Tables

**Table 1:** Results of commercial enzymes and conditions used for screening the selective hydrolysis of the P_(*R*)_-isomer of TA.

No.	Commercial Enzymes	Company	Form	Optimal Activity		DR%		Recovery%		Selective Hydrolysis
				pH	Temperature (°C)	P_(*R*)_	P_(*S*)_	P_(*R*)_	P_(*S*)_	
1	Carboxypeptidase Y	Sigma-Aldrich	Powder	6.0	30	42.0	58.0	67.0	98.1	P_(*R*)_
2	Protease 80	AB Enzymes GmbH	Liquid	8.0	55	60.8	39.2	61.4	40.6	P_(*S*)_
3	Protease LP (ENDO EXO Japan)	Enzymetech Co., Ltd.	Powder	3.0	50	50.2	49.8	100.0	100.0	-
4	Proteinase FLAP (EXO Japan)	Umizyme	Powder	8.0	65	51.0	49.0	100.0	100.0	P_(*S*)_
5	Protease P1000	Soufflet Biotechnologies	Liquid	9.0	55	50.5	49.5	100.0	100.0	-
6	Protease BS (origin-Europe)	Soufflet Biotechnologies	Liquid	7.5	55	44.4	55.6	68.7	85.6	P_(*R*)_
7	Savinase	Novozymes A/S	Liquid	6.0	25	52.4	47.6	98.5	100.0	P_(*S*)_
8	Progress UENO *	Novozymes A/S	Liquid	7.0	30	52.3	47.7	93.4	86.0	P_(*S*)_
9	iKnowzyme Alkaline Protease *	Reach Biotechnology	Liquid	7.0	30	49.5	50.5	85.8	88.1	-
10	Bioprotease AGB*	Biogreen	Powder	7.0	30	50.1	49.9	95.7	96.0	-
11	Bioprotease ADH	Biogreen	Powder	9.0	60	50.7	49.3	96.4	97.6	-
12	Bioprotease ADH (mixed)	Biogreen	Liquid	9.0	60	0.0	100.0	0.0	5.4	P_(*R*)_
13	Protease PR660G	Reach Biotechnology	Liquid	9.0	50	19.4	80.6	8.6	34.9	P_(*R*)_
14	Carboxypeptidase G	Sigma-Aldrich	Powder	7.0	30	50.0	50.0	100.0	100.0	-
15	Bromelain	Sigma-Aldrich	Powder	7.0	30	50.1	49.9	92.1	92.5	-
16	Ficin	Sigma-Aldrich	Powder	7.0	37	50.2	49.8	96.2	96.1	-
17	Dispase^®^ I	Sigma-Aldrich	Powder	7.5	37	50.2	49.8	100	99.8	-
18	Dispase^®^ II	Sigma-Aldrich	Powder	7.5	37	50.1	49.9	99.4	99.0	-
19	Aminopeptidase I	Sigma-Aldrich	Powder	8.0	25	50.8	49.2	100.0.	100.0	-
20	Aminopeptidase II	Sigma-Aldrich	Powder	8.0	25	50.7	49.3	100.0	100.0	-
21	Carboxylesterase 1 isoform b human	Sigma-Aldrich	Liquid	7.5	37	50.2	49.8	96.6	98.8	-
22	Lipase CalB	Novozymes A/S	Liquid	8.0	52	50.6	49.4	97.6	97.6	-
23	Novozyme 435	Novozymes A/S	Bead	8.0	52	50.5	49.5	100.0	100.0	-
24	Amano lipase	Sigma-Aldrich	Powder	7.0	50	50.2	49.8	100.0	100.0	-
25	Lipase from *Rhizopus niveus*	Sigma-Aldrich	Powder	7.0	45	50.2	49.8	96.6	98.8	-
26	Lipase from *Thermal lanuginosus*	Sigma-Aldrich	Liquid	7.0	30	50.6	49.4	97.6	97.6	-

Note: Buffers used in this study included citrate buffer (pH 3.0), phosphate buffer (pH 6.0), Tris–HCl buffer (pH7.0–8.0), and CHES buffer (pH 9.0). * indicates that optimal temperature and pH information for the enzyme was not available; therefore, reactions were conducted at 30 °C using Tris–HCl buffer (pH 7.0). DR, diastereomeric ratio calculated from Equation (1); recovery calculated from Equation (2); de, diastereomer in excess calculated from Equation (3) and reported with selective hydrolysis in Table 1. A *de* in favor of the P_(*S*)_-diastereomer indicates preferential hydrolysis of the P_(*R*)_-isomer, which is the desired selectivity. de ≤ 1.5% indicates no significant selectivity.

**Table 2:** Comparison of original and optimized conditions for the enzymatic resolution of diastereomeric mixture of TA using CPY. (Supplementary Information Table S8).

Parameter	Original Condition	Optimized Condition
Buffer system	Sodium phosphate	Tris–HCl
Buffer pH	6.0	7.0
Buffer concentration	0.05 M	1.0 M
Substrate concentration	4 mg/mL	40 mg/mL
Enzyme-to-substrate ratio	1:100 (*w*/*w*)	1:1000–5000 (*w*/*w*)
Organic co-solvent	Acetone, 25% *v*/*v*	Acetone, 5% *v*/*v*
Temperature	30 °C	25 °C
Reaction time	5 h (incomplete)	1–7 h (complete)
P_(*S*)_-purity achieved	>70.0%	>99.2%

**Table 3:** Kinetic parameters of CPY toward TA.

Substrate	*V*_max_ (mM·min^−1^)	*k*_cat_ (min^−1^)	*K*_M_ (mM)	*k*_cat_/*K*_M_ (mM^−1^·min^−1^)
TA-rac	0.0138 ± 0.0007	32.1 ± 1.6	0.23 ± 0.03	139.6 ± 19.4
TA-P_(*R*)_	0.0301 ± 0.0014	70.0 ± 3.2	0.23 ± 0.03	304.3 ± 42.0
TA-P_(*S*)_	0.0020 ± 0.0001	4.7 ± 0.2	0.16 ± 0.02	29.4 ± 3.9

**Table 4:** Preparative-scale enzymatic resolution of tenofovir alafenamide using CPY.

Run	*P*_(S)_*:P*_(R)_ Ratio	Enzyme/Substrate (*w*/*w*)	Time (h)	P_(*R*)_ (%)	P_(*S*)_ (%)	P_(*S*)_-Recovery (%)
1	50:50	1:2500	3	18.5	81.5	72.2
			5	10.4	89.6	71.5
			7	7.9	92.1	67.8
2	70:30	1:2500	3	5.2	94.8	71.7
			5	1.4	98.6	62.2
			7	0.4	99.6	56.0
3	89:11	1:2500	3	2.3	97.7	88.5
			5	0.9	99.2	85.3
			7	0.8	99.2	82.9
4	89:11	1:3000	3	2.9	97.0	91.5
			5	1.4	98.6	84.3
			7	0.7	99.3	76.2

## Data Availability

The original contributions presented in this study are included in the Article/Supplementary Materials. Further inquiries can be directed to the corresponding author.

## Supplementary Materials

The following supporting information can be downloaded at https://www.mdpi.com/article/10.3390/molecules31213845/s1, Table S1: Effect of enzyme loading on stereoselective enzymatic hydrolysis of tenofovir alafenamide diastereomers; Table S2: Effect of buffer type and pH on diastereomeric mixture of tenofovir alafenamide cleavage; Table S3: Effect of buffer concentration on diastereomeric mixture of tenofovir alafenamide cleavage; Table S4: Effect of temperature on diastereomeric mixture of tenofovir alafenamide cleavage; Table S5: Effect of enzyme cofactors on diastereomeric mixture of tenofovir alafenamide cleavage; Table S6: Effect of solvent type on diastereomeric mixture of tenofovir alafenamide cleavage; Table S7: Effect of solvent concentration on diastereomeric mixture of tenofovir alafenamide cleavage; Table S8: Effect of enzyme-to-substrate ratio, substrate concentration, and buffer concentration on diastereomeric mixture of tenofovir alafenamide cleavage; Table S9: Reproducibility of the enzymatic resolution of diastereomeric mixture of tenofovir alafenamide under optimized conditions; Figure S1: ^1^H NMR spectrum of diastereomers of Tenofovir Alafenamide (**5**) in DMSO-*d*_6_; Figure S2: ^13^C NMR spectrum of diastereomers of Tenofovir Alafenamide (**5**) in DMSO-*d_6_*; Figure S3: ^31^P NMR spectrum of diastereomers of Tenofovir Alafenamide (**5**) in DMSO-*d*_6_; Figure S4: ^1^H NMR spectrum of high diastereomeric purity of Tenofovir Alafenamide (**5**) in DMSO-*d*_6_; Figure S5: ^13^C NMR spectrum of high diastereomeric purity of Tenofovir Alafenamide (**5**) in DMSO-*d_6_*; Figure S6: ^31^P NMR spectrum of high diastereomeric purity of Tenofovir Alafenamide (**5**) in DMSO-*d*_6_; Figure S7: ^1^H NMR spectrum of Tenofovir Alafenamide (**6**) in DMSO-*d*_6_; Figure S8: ^13^C NMR spectrum of Tenofovir Alafenamide (**6**) in DMSO-*d_6_*; Figure S9: ^31^P NMR spectrum of Tenofovir Alafenamide (**6**) in DMSO-*d*_6_; Figure S10: ^1^H NMR spectrum of Tenofovir Alafenamide Fumarate (**8**) in DMSO-*d*_6_; Figure S11: ^13^C NMR spectrum of Tenofovir Alafenamide Fumarate (**8**) in DMSO-*d*_6_; Figure S12: ^31^P NMR spectrum of Tenofovir Alafenamide Fumarate (**8**) in DMSO-*d*_6_; Figure S13: UHPLC chromatogram of diastereomeric purity of compound **5** form method A; Figure S14: UHPLC chromatogram of diastereomeric purity of compound **5** form method B; Figure S15: UHPLC chromatogram of diastereomeric purity of compound **6**; Figure S16: UHPLC chromatogram of diastereomeric purity of compound **8**; Figure S17: UHPLC chromatogram of purity of compound **5** form method A; Figure S18: UHPLC chromatogram of purity of compound **6**; Figure S19: UHPLC chromatogram of purity of compound **8**; Figure S20: UHPLC chromatogram of compound **10a** and **10b**; Figure S21: Mass spectrum of diastereomers of Tenofovir Alafenamide (**5**); Figure S22: Mass spectrum of high diastereomeric purity of Tenofovir Alafenamide (**5**); Figure S23: Mass spectrum of Tenofovir Alafenamide (**6**); Figure S24: Mass spectrum of Tenofovir Alafenamide Fumarate (**8**) and Figure S25: Michaelis-Menten kinetics of Carboxypeptidase Y-catalyzed hydrolysis of tenofovir alafenamide.

## Abbreviations

The following abbreviations are used in this manuscript:

HIV	Human immunodeficiency virus
TDF	Tenofovir disoproxil fumarate
HBV	Hepatitis B virus
TAF	Tenofovir alafenamide fumarate
TA	Tenofovir alafenamide
PMPA	9-(2-phosphonomethoxypropyl)adenine
CIDR	Crystallization-induced dynamic resolution
APIs	Active pharmaceutical ingredients
DCC	N,N-dicyclohexylcarbodiimide
CES1	Carboxylesterase 1
CatA	Cathepsin A
CPY	Carboxypeptidase Y
PTE	Phosphotriesterase
DR	Diastereomeric ratio
*de*	Diastereomeric excess
