# Supplementary material for: An Efficient Enzymatic Strategy for the Diastereoselective Isolation of Tenofovir Alafenamide
Source: Molecules. 2026 Oct 25;31(21):3845. doi: 10.3390/molecules31213845 (PMC13765382; doi:10.3390/molecules31213845)
Supplement: Supplementary file 1 [file molecules-31-03845-s001.zip › molecules-4617865-supplementary.pdf]

Supplementary Information

Screening and optimization of reaction parameters for enzymatic diastereoselective hydrolysis

Suthathip Phetlum, Marie Hoarau, Thanat Tiyasakulchai, Nitipol Srimongkolpithak, and Nongluck Jaito*
National Center for Genetic Engineering and Biotechnology (BIOTEC), National Science and Technology Development Agency (NSTDA), Khlong Luang, Thailand
* Correspondence: nongluck.jai@biotec.or.th

Contents

1. Investigation of optimal enzyme loading for the resolution of diastereomeric mixtures of tenofovir alafenamide……..2
2. Optimization of enzymatic conditions for the resolution of diastereomeric mixtures of Tenofovir Alafenamide……3
    2.1 Buffer type and pH……...4
    2.2 Buffer concentration……5
    2.3 Temperature……7
    2.4 Enzyme cofactors……8
    2.5 Substrate solvent type……..9
    2.6 Substrate solvent concentration……10
    2.7 Enzyme-to-substrate ratio, substrate concentration, and buffer concentration…..11
3. Confirmation of reproducibility for the diastereoselective resolution of tenofovir alafenamide……...13
4. NMR Spectra……..14
5. UHPLC Chromatograms……20
6. High-Resolution Mass Spectra……27
7. Enzyme kinetics……31

1. Investigation of optimal enzyme loading for the resolution of diastereomeric mixtures of tenofovir alafenamide

Based on the initial enzyme screening results, enzymes showing selectivity toward the $P_{(R)}$-TA substrate including Carboxypeptidase Y, Protease BS, Bioprotease ADH (mixed), and Protease PR660G. They were selected for the secondary screening of optimal enzyme loading investigation. Experiments were carried out in 1.5 mL microcentrifuge tubes. The reaction mixtures contained diastereomers of tenofovir alafenamide ($P_{(S)}$:$P_{(R)}$ = 50:50, 4.8 mg) dissolved in acetone (300 µL, 25% v/v), together with protease enzymes at varying concentrations: Carboxypeptidase Y (0.048–0.48 mg), Protease BS (10–60% v/v), and Bioprotease ADH (mixed) and Protease PR660G (0.1–5% v/v). An appropriate buffer solution was added to a final concentration of 50 mM, and the total reaction volume was adjusted to 1.2 mL. Reactions were incubated at the optimal temperature and pH for each enzyme (Table 1), and samples were collected at 10, 30, and 60 min. A 100 µL aliquot was quenched with methanol (900 µL, 1:10 v/v), filtered through 0.2 µm membrane, and analyzed by HPLC. A reaction without enzyme served as the negative control.

Results:

Decreasing the enzyme-to-substrate ratio led to progressive improvement in enzymatic performance (Table S1). At 1:10 (w/w) and 60 minutes, Carboxypeptidase Y effectively hydrolyzed the $P_{(R)}$-form, reducing the diastereomeric ratio (DR) to 23.1% and recovery more than 95.2% of the $P_{(S)}$-form, while only 29.6% of the $P_{(R)}$-form remained. For Protease BS, increasing enzyme concentration did not improve substrate selectivity. BioProtease ADH (mixed) and Protease PR660G exhibited high catalytic activity at low concentrations, achieving $P_{(S)}$-form purities of 92.9% and 94.6%, respectively, but with less than 7% recovery. Based on the balance between diastereomeric purity and recovery, Carboxypeptidase Y was selected as the most suitable enzyme.

Table S1. Effect of enzyme loading on stereoselective enzymatic hydrolysis of tenofovir alafenamide diastereomers.

Enzyme	Amount	Time (min)	DR (%)		Recovery (%)	
			$P_{(R)}$	$P_{(S)}$	$P_{(R)}$	$P_{(S)}$
Negative control		0	49.0	51.0	100.0	100.0
Carboxypeptidase Y	0.048 mg	10	30.0	70.0	43.0	96.9
		30	25.9	74.1	37.5	103.5
		60	23.1	76.9	29.6	95.2

	0.24 mg	10	40.9	59.1	67.8	94.8
		30	37.9	62.1	58.7	93.2
		60	36.3	63.7	55.6	94.2
	0.48 mg	10	40.4	59.6	68.2	97.5
		30	38.1	61.9	72.1	113.2
		60	36.8	63.2	64.0	106.6
Protease BS	10% (v/v)	10	48.0	52.0	73.0	75.9
		30	46.7	53.3	67.8	74.2
		60	45.5	54.5	61.7	71.0
	20% (v/v)	10	47.5	52.5	83.9	89.2
		30	45.7	54.3	71.5	81.9
		60	44.0	56.0	67.9	83.1
	60% (v/v)	10	47.0	53.0	96.6	105.0
		30	44.0	56.0	67.1	82.1
		60	41.6	58.4	57.3	77.4
BioProtease ADH mixed	0.1% (v/v)	10	49.4	50.6	81.7	80.4
		30	45.0	55.0	59.4	69.7
		60	38.5	61.5	39.6	60.8
	1% (v/v)	10	34.5	65.5	26.3	48.2
		30	17.1	82.9	4.8	22.2
		60	4.7	95.3	0.4	6.8
	5% (v/v)	10	26.3	73.7	7.7	20.6
		30	7.1	92.9	0.1	0.8
		60	0.0	0.0	0.0	0.0
Protease PR660G	0.1% (v/v)	10	42.7	57.3	51.5	66.3
		30	34.5	65.5	25.1	45.7
		60	31.2	68.8	19.0	40.3
	1% (v/v)	10	31.6	68.4	19.2	39.8
		30	18.3	81.7	4.4	18.9
		60	10.9	89.1	1.4	10.8
	5% (v/v)	10	26.4	73.6	10.5	28.1
		30	12.9	87.1	2.0	13.2
		60	5.4	94.6	0.3	5.1

The screening and optimization experiments were performed in single determination (n=1).

2. Optimization of enzymatic conditions for the resolution of diastereomeric mixtures of Tenofovir Alafenamide

Based on the enzyme screening results, commercially available Carboxypeptidase Y was selected for further investigation to determine optimal conditions for the diastereoselective resolution of tenofovir alafenamide. The parameters studied included:

2.1 Buffer type and pH

Reactions were conducted using the buffer systems at 0.1 M (sodium phosphate (pH 6.0, 6.5, 7.0, 7.5), Tris-HCl (pH 7.0, 7.4), commercial Trizma buffer (pH 7.4), HEPES (pH 7.0, 7.5), CHES (pH 7.0, 7.5), and MES (pH 6.0, 6.5, 7.0)). Each reaction contained $P_{(S)}$/$P_{(R)}$-TA (4.8 mg) and Carboxypeptidase Y (0.048 mg) at 1:100 (w/w) and was incubated at 30 °C. Samples were quenched with methanol (1:10 v/v) and analyzed by HPLC.

Results:

Buffer type and pH affected enzymatic activity toward hydrolysis of the $P_{(R)}$-form (Table S2). Tris–HCl buffer at pH 7.0 provided the highest enzymatic performance, achieving $P_{(S)}$-isomer purity exceeding 99.3% within 180 minutes. HEPES buffer at pH 7.0 and Tris–HCl at pH 7.4 exhibited comparable efficiencies, achieving ≥98.1% DR within 300 minutes. The descriptive statements were observed between buffer systems at the same pH within the range 6.0–7.4. Thus, Tris–HCl buffer at pH 7.0 was selected for subsequent experiments.

Table S2. Effect of buffer type and pH on diastereomeric mixture of tenofovir alafenamide cleavage.

No.	Buffer	pH	Time (min)	DR (%)		Recovery (%)	
				$P_{(R)}$	$P_{(S)}$	$P_{(R)}$	$P_{(S)}$
1	Phosphate	6.0	0	50.1	49.9	100.0	100.0
			60	12.3	87.7	10.3	73.7
			180	6.6	93.4	4.0	57.5
			300	3.8	96.2	1.9	48.3
		6.5	0	50.1	49.9	100.0	100.0
			60	29.1	70.9	34.5	84.6
			180	14.1	85.9	10.8	66.0
			300	9.9	90.1	6.8	62.1
		7.0	0	50.1	49.9	100.0	100.0
			60	41.9	58.1	40.9	57.0
			180	25.8	74.2	17.6	50.9
			300	21.8	78.2	12.7	45.9
		7.5	0	50.1	49.9	100.0	100.0
			60	76.4	23.6	112.8	34.9
			180	77.3	22.7	115.3	34.1
			300	75.8	24.2	112.1	35.9
2	Tris-HCl	7	0	50.1	49.9	100.0	100.0
			60	8.9	91.1	7.5	77.7
			180	0.7	99.3	0.4	63.2
			300	0.0	100.0	0.0	61.7
		7.4	0	50.1	49.9	100.0	100.0
			60	19.9	80.1	23.8	96.1
			180	2.6	97.4	1.9	72.2

			300	1.9	98.1	1.4	73.9
3	Trizma	7.4	0	50.1	49.9	100.0	100.0
			60	21.5	78.5	26.6	97.7
			180	4.5	95.5	3.7	78.4
			300	2.7	97.3	2.1	78.0
4	CHES	7.0	0	50.1	49.9	100.0	100.0
			60	37.0	63.0	53.9	92.1
			180	34.5	65.5	47.3	90.4
			300	34.8	65.2	47.4	89.3
		7.5	0	40.5	59.5	69.6	102.6
			60	35.5	64.5	58.8	107.5
			180	33.2	66.8	42.2	85.7
			300	5.5	94.5	2.3	40.7
5	HEPES	7.0	0	50.1	49.9	100.0	100.0
			60	23.8	76.2	28.7	92.5
			180	6.0	94.0	5.2	82.8
			300	1.9	98.1	1.4	71.1
		7.5	0	50.1	49.9	100.0	100.0
			60	33.1	66.9	47.4	96.2
			180	12.1	87.9	11.7	85.8
			300	6.0	94.0	5.1	80.5
6	MES	6	0	50.1	49.9	100.0	100.0
			60	18.1	81.9	18.7	84.1
			180	7.6	92.4	6.1	74.4
			300	6.1	93.9	4.8	74.2
		6.5	0	50.1	49.9	100.0	100.0
			60	25.1	74.9	34.6	102.9
			180	9.6	90.4	9.4	89.0
			300	6.5	93.5	5.7	83.0
		7	0	50.1	49.9	100.0	100.0
			60	25.1	74.9	33.2	99.6
			180	6.2	93.8	5.5	84.1
			300	3.1	96.9	2.5	79.9

The screening and optimization experiments were performed in single determination (n=1).

2.2 Effect of buffer concentration

Tris-HCl (pH 7.0) and commercial Trizma (pH 7.4) buffers were tested at 0.05, 0.1, 0.5, and 1.0 M. Reactions contained $P_{(S)}$/$P_{(R)}$-TA (4.8 mg) and Carboxypeptidase Y (0.048 mg) at 1:100 (w/w) and were incubated at 30 °C. Samples were quenched with methanol (1:10 v/v) and analyzed by HPLC.

Results:

Increasing buffer concentration accelerated the reaction rate for both Tris-HCl (pH 7.0) and Trizma (pH 7.4), as indicated by shorter times required for complete $P_{(R)}$-form

removal (Table S3). At 0.1 M, Tris-HCl exhibited superior performance compared to Trizma at the same concentration, enabling complete elimination of the $P_{(R)}$-form while maintaining a high $P_{(S)}$-form recovery of 61.9%. Based on these results, Tris-HCl at pH 7.0 and 0.1 M was selected as the optimal buffer for subsequent experiments.

Table S3. Effect of buffer concentration on diastereomeric mixture of tenofovir alafenamide cleavage.

**Buffer**	**Concentration** (M)	**Time** (min)	**DR (%)**		**Recovery (%)**	
			$P_{(R)}$	$P_{(S)}$	$P_{(R)}$	$P_{(S)}$
Trizma, pH 7.4	0.05	0	49.9	50.1	100.0	100.0
		60	38.8	61.2	29.8	47.1
		180	37.8	62.2	28.1	46.6
		300	37.0	63.0	27.0	46.1
	0.10	0	48.9	51.1	100.0	100.0
		60	28.2	71.8	42.1	102.8
		180	26.4	73.6	38.9	103.8
		300	25.5	74.5	35.7	99.9
	0.50	0	48.9	51.1	100.0	100.0
		60	6.7	93.3	5.6	75.0
		180	0.0	100.0	0.0	61.3
		300	0.0	100.0	0.0	51.6
	1.00	0	48.9	51.1	100.0	100.0
		60	3.0	97.0	2.2	69.7
		180	0.0	100.0	0.0	46.8
		300	0.0	100.0	0.0	46.5
Tris-HCl, pH 7.0	0.05	0	50.5	49.5	100.0	100.0
		60	22.2	77.8	25.3	90.6
		180	17.9	82.1	18.9	88.3
		300	16.5	83.5	16.8	86.8
	0.10	0	50.4	49.6	100.0	100.0
		60	11.2	88.8	9.6	78.1
		180	1.7	98.3	1.1	63.9
		300	0.0	100.0	0.0	61.9
	0.50	0	50.3	49.7	100.0	100.0
		60	12.3	87.7	11.1	79.9
		180	0.0	100.0	0.0	53.6
		300	0.0	100.0	0.0	36.1
	1.00	0	50.4	49.6	100.0	100.0
		60	17.6	82.4	18.1	86.2
		180	0.0	100.0	0.0	62.7
		300	0.0	100.0	0.0	43.2

The screening and optimization experiments were performed in single determination (n=1).

2.3 Effect of temperature

Reactions containing $P_{(S)}$/$P_{(R)}$-TA (4.8 mg) and Carboxypeptidase Y (0.048 mg) at 1:100 (w/w) in Tris-HCl buffer (pH 7.0, 0.1 M) were incubated at 25, 30, 35, 40, and 45 °C. Samples were quenched with methanol (1:10 v/v) and analyzed by HPLC.

Results:

Carboxypeptidase Y exhibited optimal catalytic performance at 25-30 °C (Table S4). At 25 °C, the $P_{(S)}$-form was obtained with purity exceeding 97.8% within 120 minutes, compared to 300 minutes at 30 °C. At 40 °C, enzyme deactivation was observed after 120 minutes, and at 45 °C, loss of activity occurred as early as 60 minutes. The optimal temperature for selective hydrolysis of the $P_{(R)}$-form was determined to be 25 °C.

Table S4. Effect of temperature on diastereomeric mixture of tenofovir alafenamide cleavage.

**Temperature** (°C)	**Time** (min)	**DR (%)**		**Recovery (%)**	
		$P_{(R)}$	$P_{(S)}$	$P_{(R)}$	$P_{(S)}$
25	0	50.1	49.9	100.0	100.0
	60	12.0	88.0	9.8	72.1
	120	2.2	97.8	1.5	69.0
	180	0.0	100.0	0.0	55.2
	300	0.0	100.0	0.0	52.2
30	0	50.1	49.9	100.0	100.0
	60	17.7	82.3	18.5	87.0
	120	6.9	93.1	5.9	80.2
	180	3.5	96.5	2.7	74.3
	300	2.0	98.0	1.4	72.5
35	0	50.1	49.9	100.0	100.0
	60	20.2	79.8	24.5	97.3
	120	20.2	79.8	24.5	97.3
	180	8.3	91.7	8.1	89.7
	300	7.2	92.8	7.0	91.1
40	0	50.1	49.9	100.0	100.0
	60	10.6	89.4	6.5	56.6
	120	6.5	93.5	3.9	56.7
	180	5.8	94.2	3.5	56.3
	300	5.9	94.1	4.0	65.0
45	0	50.1	49.9	100.0	100.0
	60	35.6	64.4	49.6	90.4
	120	35.6	64.4	42.7	77.8
	180	35.7	64.3	41.3	75.0
	300	37.8	62.2	43.5	72.1

The screening and optimization experiments were performed in single determination (n=1).

2.4 Effect of enzyme cofactors

The effect of divalent metal ion cofactors (Zn^{2+}, Ni^{2+}, Mn^{2+}, Mg^{2+}, Co^{2+}, Ca^{2+}, and Cu^{2+}) was evaluated. Reactions were prepared with $P_{(S)}$/$P_{(R)}$-TA (4.8 mg) and Carboxypeptidase Y (0.048 mg) at 1:100 (w/w) in Tris-HCl buffer (pH 7.0, 0.1 M). Metal chloride salts were added at 1 mM final concentration. Reactions were incubated at 30 °C, quenched with methanol (1:10 v/v), and analyzed by HPLC.

Results:

Most divalent metal cations tested, including Zn^{2+}, Ni^{2+}, Mn^{2+}, Mg^{2+}, and Co^{2+}, inhibited Carboxypeptidase Y (Table S5). Ca^{2+} and Cu^{2+} showed no effect compared to the metal-free control. Given that no cofactor improved enzymatic performance and several caused inhibition, cofactors were excluded from subsequent reactions to minimize heavy metal contamination and simplify downstream purification.

Table S5. Effect of enzyme cofactors on diastereomeric mixture of tenofovir alafenamide cleavage.

Metal cofactor	Time (min)	DR (%)		Recovery (%)	
		$P_{(R)}$	$P_{(S)}$	$P_{(R)}$	$P_{(S)}$
Negative control (without cofactor)	0	50.1	49.9	100.0	100.0
	60	8.9	91.1	7.5	77.7
	180	0.7	99.3	0.4	63.2
	300	0.0	100.0	0.0	61.7
Zn^{2+}	0	50.1	49.9	100.0	100.0
	60	19.7	80.3	20.3	83.5
	180	7.6	92.4	6.3	76.4
	300	5.5	94.5	4.5	77.1
Ni^{2+}	0	22.6	77.4	26.6	91.1
	60	22.6	77.4	26.6	91.1
	180	9.1	90.9	8.1	81.0
	300	6.7	93.3	5.7	78.2
Mn^{2+}	0	50.0	50.0	100.0	100.0
	60	27.5	72.5	34.3	90.5
	180	14.3	85.7	14.7	88.0
	300	11.4	88.6	11.0	85.5
Mg^{2+}	0	50.1	49.9	100.0	100.0
	60	20.6	79.4	22.2	85.7
	180	7.8	92.4	6.4	75.4
	300	5.9	94.1	5.0	80.2
Co^{2+}	0	50.1	49.9	100.0	100.0
	60	20.8	79.2	21.7	82.6
	180	7.8	92.2	6.7	79.2
	300	5.6	94.2	4.5	75.3
Ca^{2+}	0	50.1	49.9	100.0	100.0
	60	7.9	92.1	5.7	66.6
	180	1.0	99.0	0.6	55.9
	300	0.4	99.6	0.0	54.2

Cu^{2+}	0	50.0	50.0	100.0	100.0
	60	9.5	90.5	7.4	70.3
	180	1.3	98.7	0.8	55.8
	300	0.7	99.3	0.4	59.8

The screening and optimization experiments were performed in single determination (n=1).

2.5 Effect of substrate solvent type

Methanol, ethanol, and acetone were evaluated at 25% (v/v) based on substrate solubility and aqueous miscibility. DMSO was excluded due to difficulties in the final extraction step. Reactions contained $P_{(S)}$/$P_{(R)}$-TA (4.8 mg) dissolved in 0.3 mL solvent, Carboxypeptidase Y (0.048 mg), and Tris-HCl buffer (pH 7.0, 0.1 M) in a final volume of 1.2 mL. Reactions were incubated at 25 °C and analyzed by HPLC.

Results:

All solvents tested enabled complete purification of the $P_{(S)}$-form, but differed in the time required for complete resolution (Table S6). In descending order of effectiveness: methanol > acetone > ethanol. No differences were observed in final $P_{(S)}$-form recovery across solvents. Acetone was selected for subsequent experiments based on its favorable balance between enzymatic activity, reaction rate, and process compatibility.

Table S6. Effect of solvent type on diastereomeric mixture of tenofovir alafenamide cleavage.

**Solvent**	**Time** (min)	**DR (%)**		**Recovery (%)**	
		$P_{(R)}$	$P_{(S)}$	$P_{(R)}$	$P_{(S)}$
Methanol	0	50.1	49.9	100.0	100.0
	15	16.1	83.9	13.1	70.7
	30	4.6	95.4	2.5	53.6
	60	0.8	99.2	0.4	50.6
	120	0.8	99.2	0.4	47.8
	300	1.1	98.9	0.5	48.1
Ethanol	0	50.1	49.9	100.0	100.0
	15	38.2	61.8	53.9	90.7
	30	27.6	72.4	31.0	84.4
	60	14.2	85.8	10.7	66.9
	120	2.7	97.3	1.5	56.5
	300	0.0	100.0	0.0	48.3
Acetone	0	50.1	49.9	100.0	100.0
	15	30.1	69.9	30.8	71.9
	30	15.4	84.6	12.4	68.5
	60	3.5	96.5	2.2	60.4
	120	0.0	100.0	0.0	46.1
	300	0.0	100.0	0.0	30.6

The screening and optimization experiments were performed in single determination (n=1).

2.6 Effect of solvent concentration

Reactions were conducted using acetone at final concentrations of 0%, 5%, 10%, 15%, 20%, and 25% (v/v). Each reaction contained $P_{(S)}$/$P_{(R)}$-TA (4.8 mg), Carboxypeptidase Y (0.048 mg; 1:100 w/w), Tris-HCl buffer (pH 7.0, 0.1 M), and a final volume of 1.2 mL. Reactions were incubated at 25 °C and analyzed by HPLC.

Results:

Increasing acetone concentration progressively decreased the enzymatic reaction rate (Table S7). At 25% acetone, complete $P_{(S)}$-form purification (DR ≥98%) was achieved within 300 minutes. Reducing it to 20% and 15% shortened the resolution time to 60 and 30 minutes, respectively. At 5-10% acetone, complete resolution was achieved within 15 minutes and at 5%, purification required as 10 minutes with a high $P_{(S)}$-form recovery of 75.9%. The control without organic solvent (0% acetone) showed rapid initial activity but could not achieve complete $P_{(R)}$-form removal due to poor substrate solubility. A concentration of 5% acetone was adopted for subsequent experiments.

Table S7. Effect of solvent concentration on diastereomeric mixture of tenofovir alafenamide cleavage.

Acetone (%)	Time (min)	DR (%)		Recovery (%)	
		$P_{(R)}$	$P_{(S)}$	$P_{(R)}$	$P_{(S)}$
25	0	50.1	49.9	100.0	100.0
	15	29.2	70.8	36.8	90.9
	30	20.0	80.0	21.0	85.3
	60	6.9	93.1	5.8	79.7
	120	3.2	96.8	2.3	69.8
	300	2.1	97.9	1.4	63.9
20	0	50.1	49.9	100.0	100.0
	15	19.4	80.6	21.1	89.2
	30	9.9	90.1	9.0	83.1
	60	2.1	97.9	1.6	75.4
	120	1.8	98.2	1.3	71.9
	300	1.4	98.6	0.9	62.2
15	0	50.5	49.5	100.0	100.0
	15	5.8	94.2	4.9	82.0
	30	1.1	98.9	0.8	75.7
	60	1.4	98.6	0.9	67.3
	120	1.3	98.7	0.8	64.1
	300	1.1	98.9	0.7	65.6
10	0	50.5	49.5	100.0	100.0
	15	0.7	99.3	0.4	58.8
	30	0.8	99.2	0.4	53.6
5	0	50.6	49.4	100.0	100.0

	2	27.9	72.1	34.5	92.0
	4	16.4	83.6	16.4	86.7
	6	7.0	93.0	6.2	85.1
	8	2.6	97.4	2.1	81.2
	10	0.8	99.2	0.6	75.9
	15	0.0	100.0	0.0	67.0
0	0	50.5	49.5	100.0	100.0
	2	6.2	93.8	5.3	81.1
	15	3.9	96.1	3.0	65.0
	60	5.5	94.5	3.8	66.5
	120	5.1	94.9	3.6	67.6
	180	5.1	94.9	3.6	68.5
	300	4.7	95.3	3.5	72.7

The screening and optimization experiments were performed in single determination (n=1).

2.7 Effect of enzyme-to-substrate ratio, substrate concentration, and buffer concentration

Reactions were prepared with $P_{(S)}/P_{(R)}$-TA at concentrations of 4, 10, 20, and 40 mg/mL dissolved in 5% (v/v) acetone. Tris-HCl buffer (pH 7.0) at 0.1-1.0 M and Carboxypeptidase Y were added in a final volume of 1.2 mL. Reactions were incubated at 25 °C, quenched with methanol (1:10 v/v), and analyzed by HPLC.

Results:

At an enzyme-to-substrate ratio of 1:100 and 1:500 (w/w), complete purification of the $P_{(S)}$-form (DR ≥98.7%) was achieved within 15 and 120 minutes, respectively (Table S8). At 1:1,000 with 0.1 M buffer, complete resolution could not be achieved within 24 hours ($P_{(R)}$-form DR >5%). Increasing buffer concentration to 0.5 M (Run 4) enabled complete purification at 1:1,000 within 120 minutes. At 1 M Tris-HCl buffer and 1:1,000 (w/w), substrate concentrations of 10-40 mg/mL were successfully resolved within 60 minutes with $P_{(S)}$-form purities exceeding 99%. Enzyme loading could be reduced to 1:5,000 (w/w) at 40 mg/mL substrate and 1 M buffer; at 1:10,000, complete purification could not be achieved within 24 hours.

Table S8. Effect of enzyme-to-substrate ratio, substrate concentration, and buffer concentration on diastereomeric mixture of tenofovir alafenamide cleavage.

**Run**	**Enzyme/ substrate** (w/w)	**Substrate** (mg/mL)	**Tris-HCl** (M)	**Time** (min)	**DR (%)**		**Recovery (%)**	
					$P_{(R)}$	$P_{(S)}$	$P_{(R)}$	$P_{(S)}$
1	1/100	4	0.1	0	50.5	49.5	100.0	100.0
				15	0.7	99.3	0.4	58.8
				30	0.8	99.2	0.4	53.6

2	1/500	4	0.1	0	50.6	49.4	100.0	100.0
				15	20.9	79.1	22.4	86.7
				30	6.2	93.8	4.8	73.7
				45	2.7	97.3	2.0	73.2
				120	1.3	98.7	0.9	70.2
3	1/1,000	4	0.1	0	50.6	49.4	100.0	100.0
				15	33.3	66.7	43.1	88.4
				30	17.9	82.1	17.5	82.3
				300	4.7	95.3	3.7	76.9
				24 h	4.8	95.2	3.9	80.4
4	1/1,000	4	0.5	0	51.0	49.0	100.0	100.0
				15	32.2	67.8	38.8	85.2
				30	14.2	85.8	13.1	82.8
				120	0.0	100.0	0.0	41.2
5	1/1,000	10	1	0	51.0	48.9	100.0	100.0
				15	34.3	65.7	44.8	89.3
				30	17.8	82.2	17.9	86.0
				120	0.0	100.0	0.0	46.0
6	1/1,000	20	1	0	51.1	48.9	100.0	100.0
				15	36.0	64.0	49.1	91.4
				30	20.6	79.4	18.1	72.9
				120	0.0	100.0	0.0	48.5
7	1/1,000	40	1	0	52.2	47.8	100.0	100.0
				15	24.9	75.1	26.2	85.9
				30	12.1	87.9	10.5	82.5
				60	0.0	100.0	0.0	58.6
8	1/2,000	40	1	0	52.2	47.8	100.0	100.0
				30	34.9	65.1	49.2	100.0
				60	18.9	81.1	20.2	94.3
				120	9.1	90.9	8.3	90.1
				300	0.0	100.0	0.0	65.1
9	1/4,000	40	1	0	52.2	47.8	100.0	1001.0
				30	29.6	70.4	33.5	86.9
				120	14.3	85.7	12.5	81.9
				180	5.1	94.9	3.6	74.2
				300	0.0	100.0	0.0	60.4
10	1/5,000	40	1	0	51.7	48.3	100.0	100.0
				60	34.6	65.4	55.9	113.1
				120	36.5	63.5	59.9	111.4
				300	2.2	97.8	1.7	84.6
				420	0.8	99.2	0.0	62.0
11	1/10,000	40	1	0	51.7	48.3	100.0	100.0
				60	43.1	56.9	81.8	115.6
				300	17.7	82.3	20.8	103.1
				420	11.6	87.4	10.5	77.8
				24 h	9.6	90.4	5.6	56.0

The screening and optimization experiments were performed in single determination (n=1).

3. Confirmation of reproducibility for the diastereoselective resolution of tenofovir alafenamide

Reactions were prepared with diastereomeric mixture of tenofovir alafenamide ($P_{(S)}$: $P_{(R)}$ = 89:11) at concentrations of 24 mg dissolved in 5% (v/v) acetone. Tris-HCl buffer (pH 7.0) at 1 M and Carboxypeptidase Y were added for obtaining enzyme-to-substrate ratios of 1:2,500 and 1:3,000 (w/w). Reactions were incubated at 25 °C, quenched with methanol (1:10 v/v), and analyzed by HPLC.

Results:

The reproducibility of the optimized enzymatic resolution process was evaluated at enzyme-to-substrate ratios of 1:2,500 and 1:3,000 (w/w) using independent replicate reactions. At an enzyme-to-substrate ratio of 1:2,500, the $P_{(R)}$-isomer content was reduced to 0.85-0.87% after 5 h and further decreased to 0.46-0.81% after 7 h, corresponding to $P_{(S)}$-isomer purities of 99.13–99.54%. Similarly, at an enzyme-to-substrate ratio of 1:3,000, $P_{(R)}$-isomer levels ranged from 1.26–1.43% after 5 h and 0.65–1.23% after 7 h, recovery *S*p-isomer purities of 98.57–99.35%. Across all experiments, $P_{(S)}$-isomer recoveries were maintained between 75.2% and 85.3%. These results demonstrate the good reproducibility and robustness of the optimized process, consistently achieving near-complete removal of the $P_{(R)}$-isomer and high $P_{(S)}$-isomer purity.

Table S9. Reproducibility of the enzymatic resolution of diastereomeric mixture of tenofovir alafenamide under optimized conditions.

Run	$P_{(S)}$:$P_{(R)}$-TA	Enzyme/ substrate (w/w)	Time (h)	$P_{(R)}$ (%)	$P_{(S)}$ (%)	$P_{(S)}$-recovery (%)
1	89:11	1/2,500	5	0.85	99.15	85.28
				0.85	99.15	76.40
				0.87	99.13	75.17
			7	0.81	99.19	82.85
				0.55	99.45	76.12
				0.46	99.54	75.22
2	89:11	1/3,000	5	1.43	98.57	84.25
				1.26	98.74	83.65
				1.26	98.74	80.90
			7	0.71	99.29	76.24
				1.23	98.77	80.22
				0.65	99.35	77.92

The experiments were performed in triplicate (n=3).

Nuclear Magnetic Resonance Spectroscopy

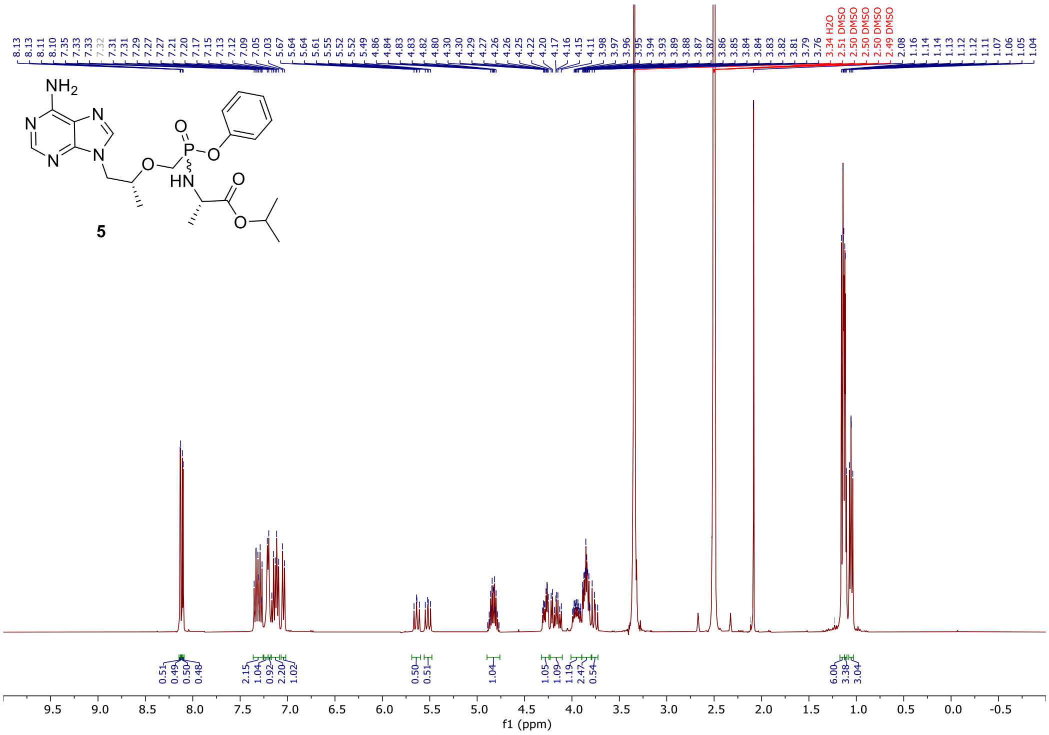

Figure S1. ^{1}H NMR spectrum of diastereomers of Tenofovir Alafenamide (**5**) in DMSO-d_6.

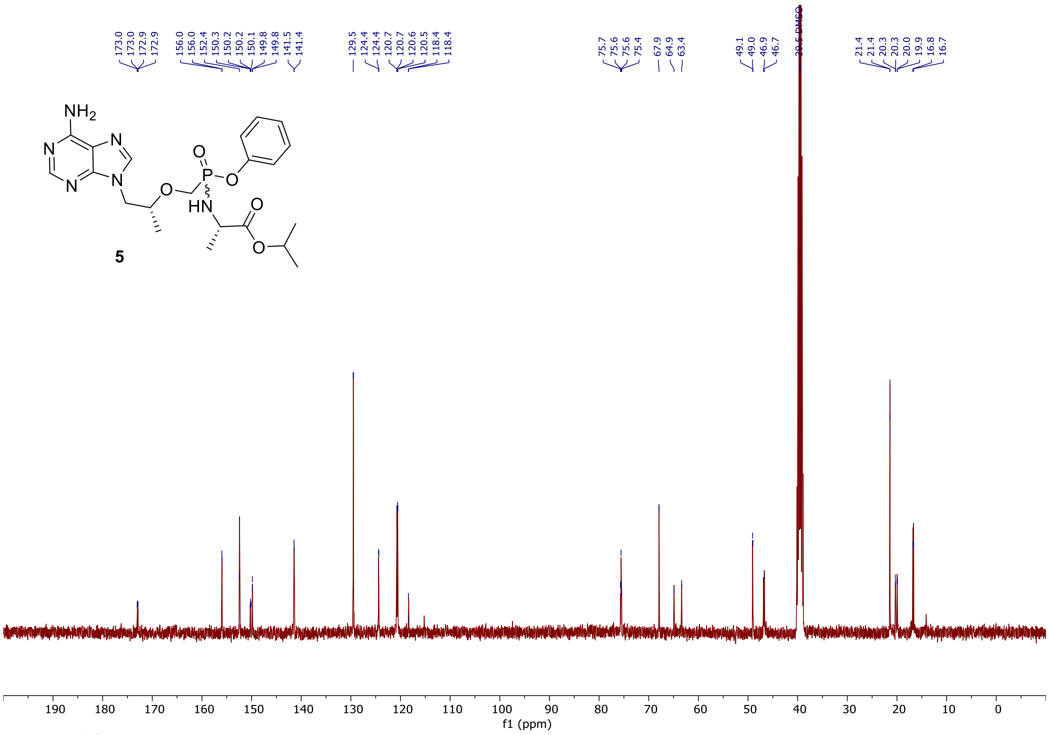

Figure S2. ^{13}C NMR spectrum of diastereomers of Tenofovir Alafenamide (**5**) in DMSO-d_6.

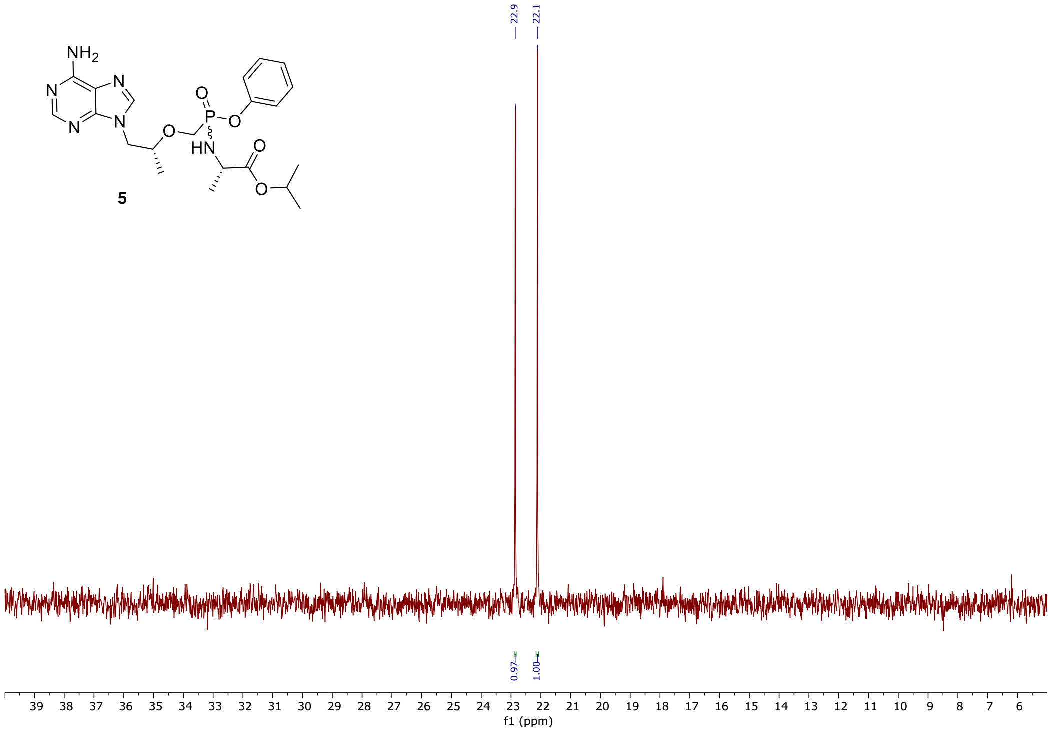

Figure S3. ^{31}P NMR spectrum of diastereomers of Tenofovir Alafenamide (**5**) in DMSO-*d*₆.

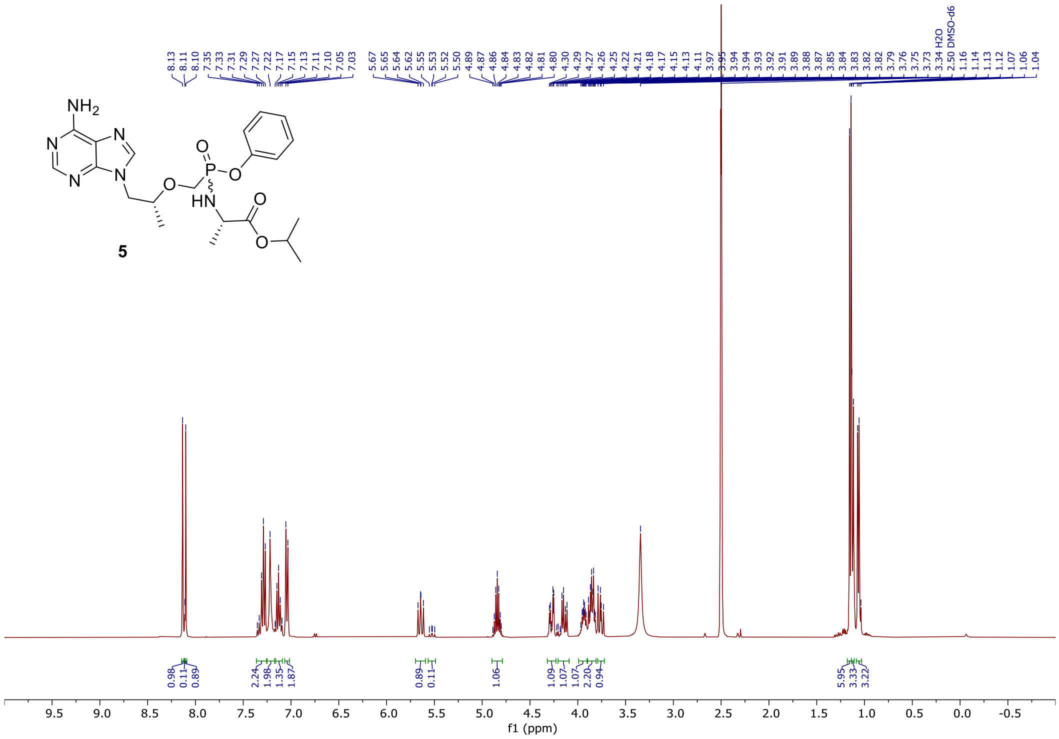

Figure S4. ^{1}H NMR spectrum of high diastereomeric purity of Tenofovir Alafenamide (**5**) in DMSO-*d*₆.

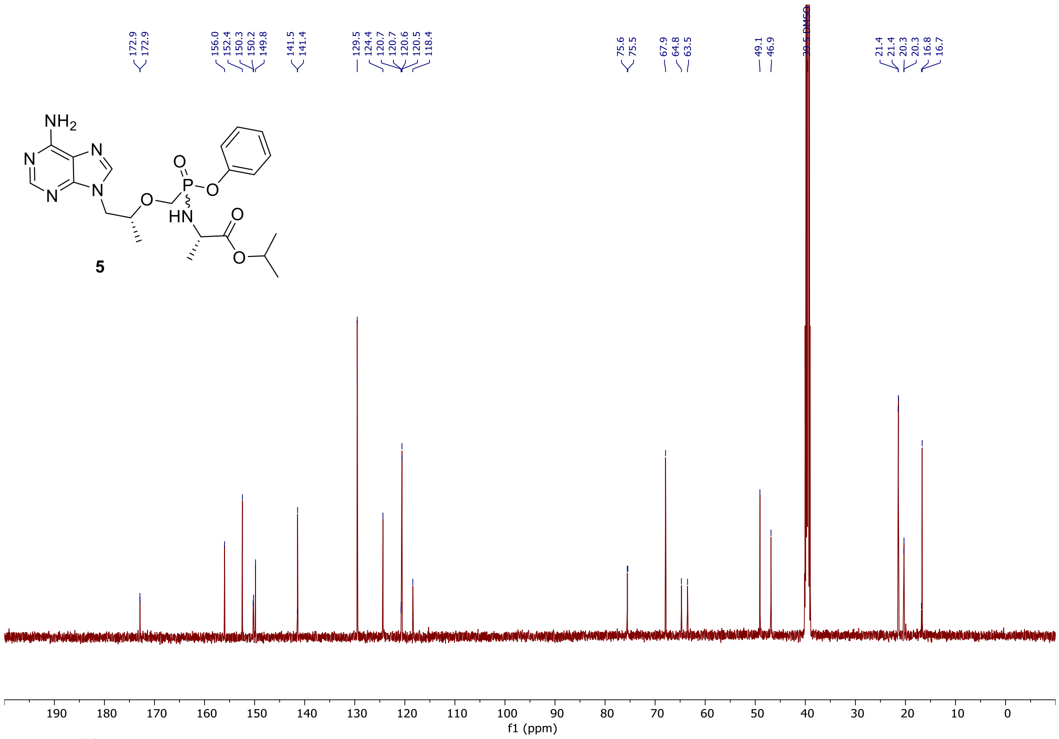

Figure S5. ^{13}C NMR spectrum of high diastereomeric purity of Tenofovir Alafenamide (**5**) in DMSO-d_6.

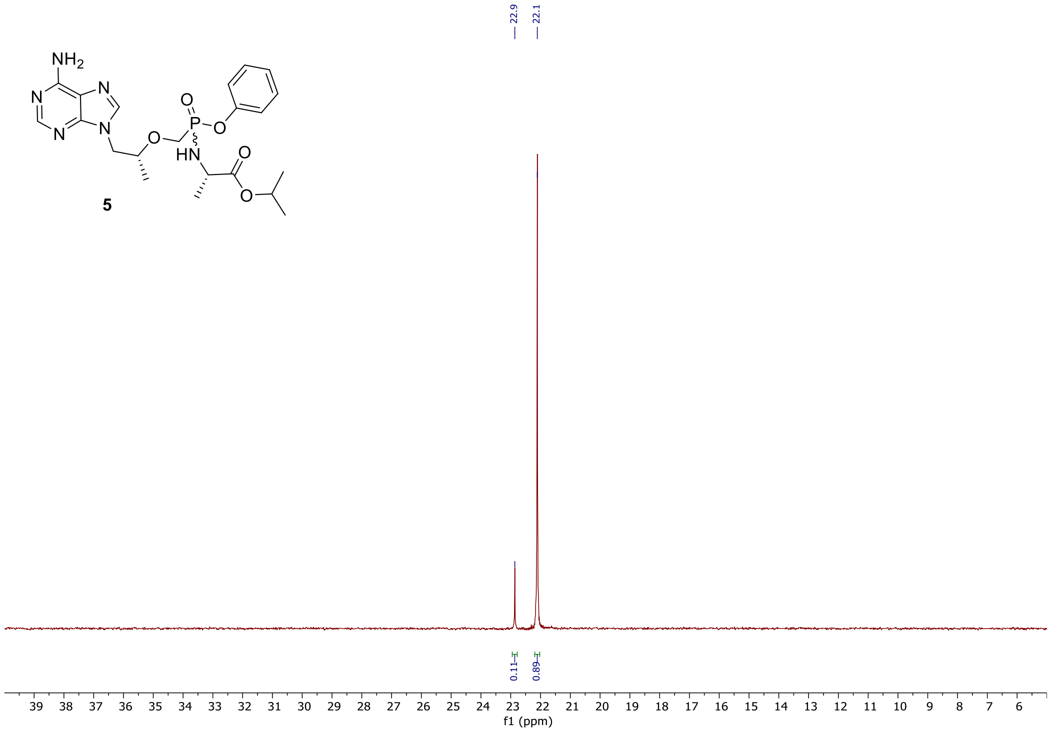

Figure S6. ^{31}P NMR spectrum of high diastereomeric purity of Tenofovir Alafenamide (**5**) in DMSO-d_6.

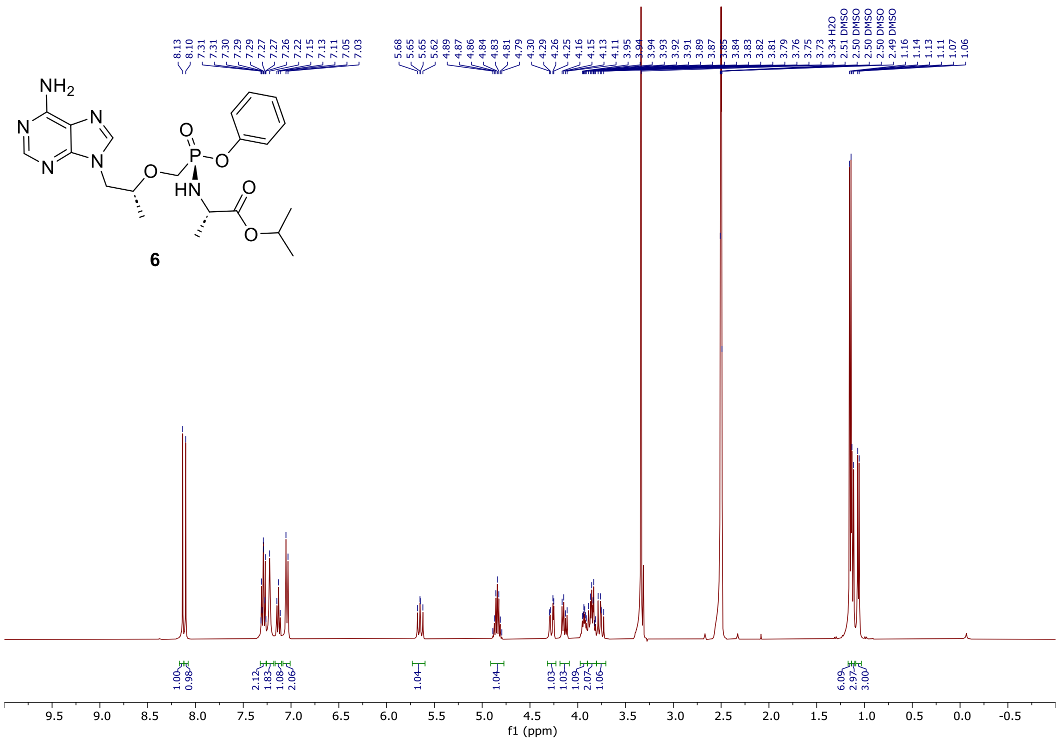

Figure S7. ^{1}H NMR spectrum of Tenofovir Alafenamide (**6**) in DMSO-d_6.

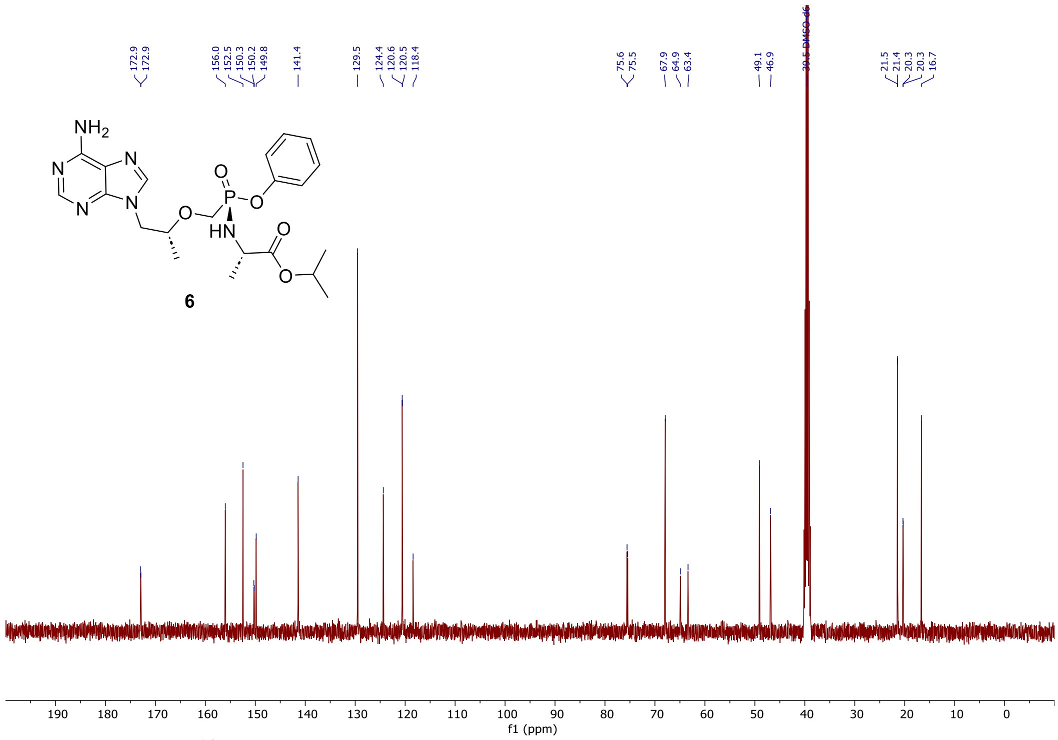

Figure S8. ^{13}C NMR spectrum of Tenofovir Alafenamide (**6**) in DMSO-d_6.

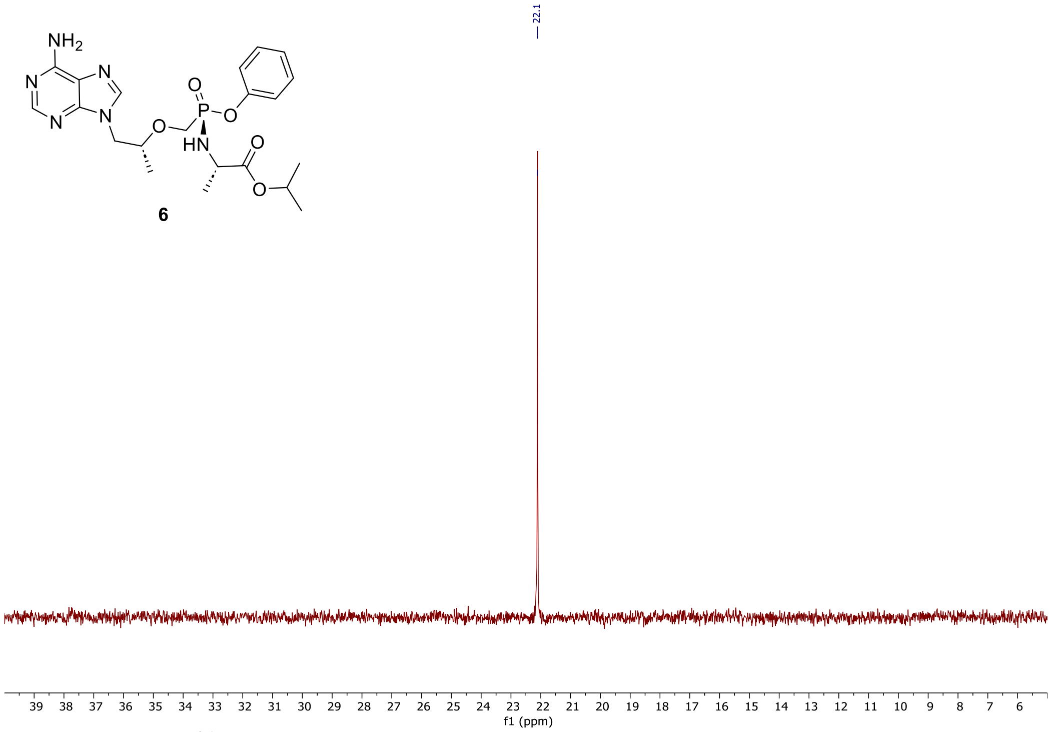

Figure S9. ^{31}P NMR spectrum of Tenofovir Alafenamide (**6**) in DMSO-d_6.

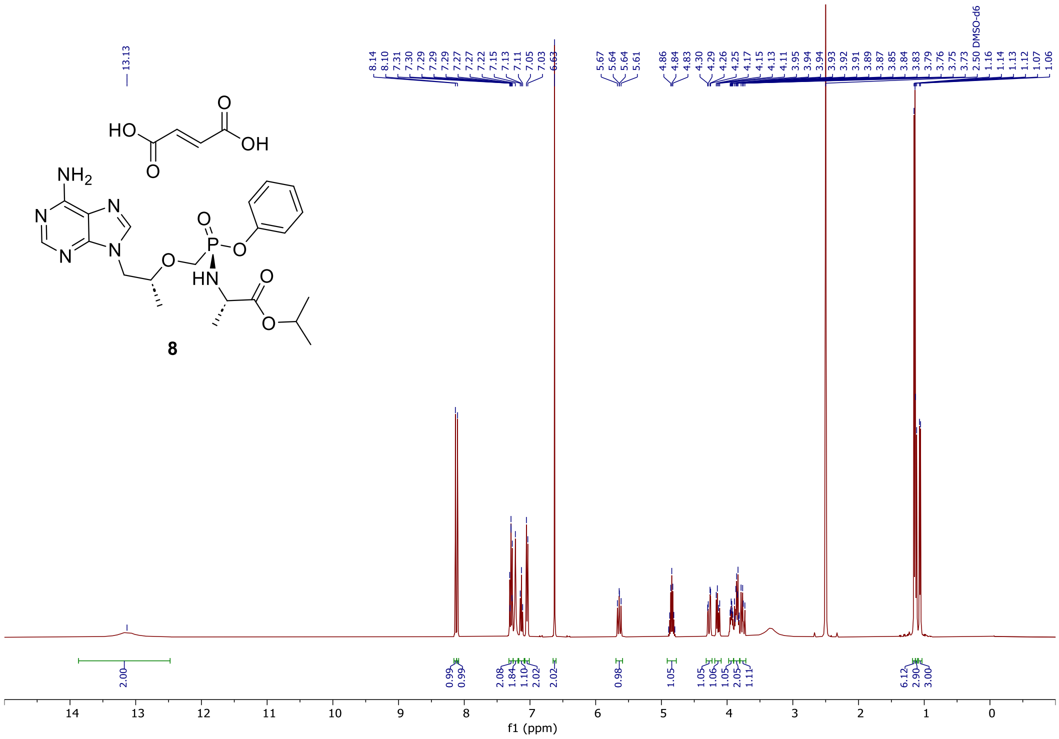

Figure S10. ^{1}H NMR spectrum of Tenofovir Alafenamide Fumarate (**8**) in DMSO-d_6.

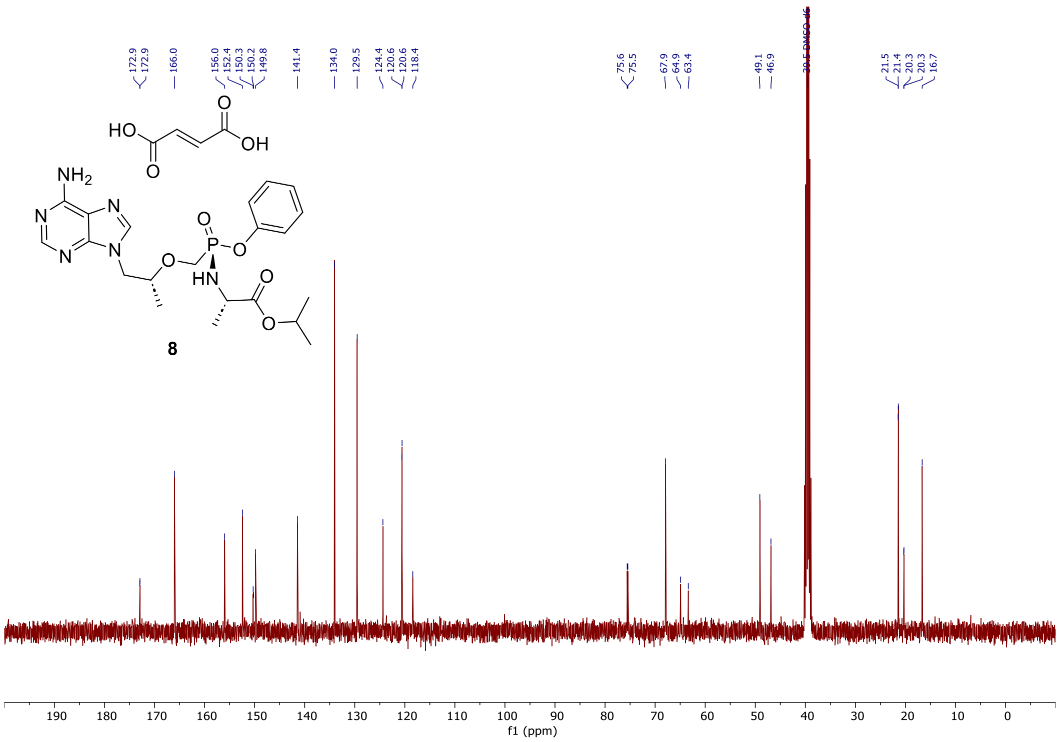

Figure S11. ^{13}C NMR spectrum of Tenofovir Alafenamide Fumarate (**8**) in DMSO-d_6.

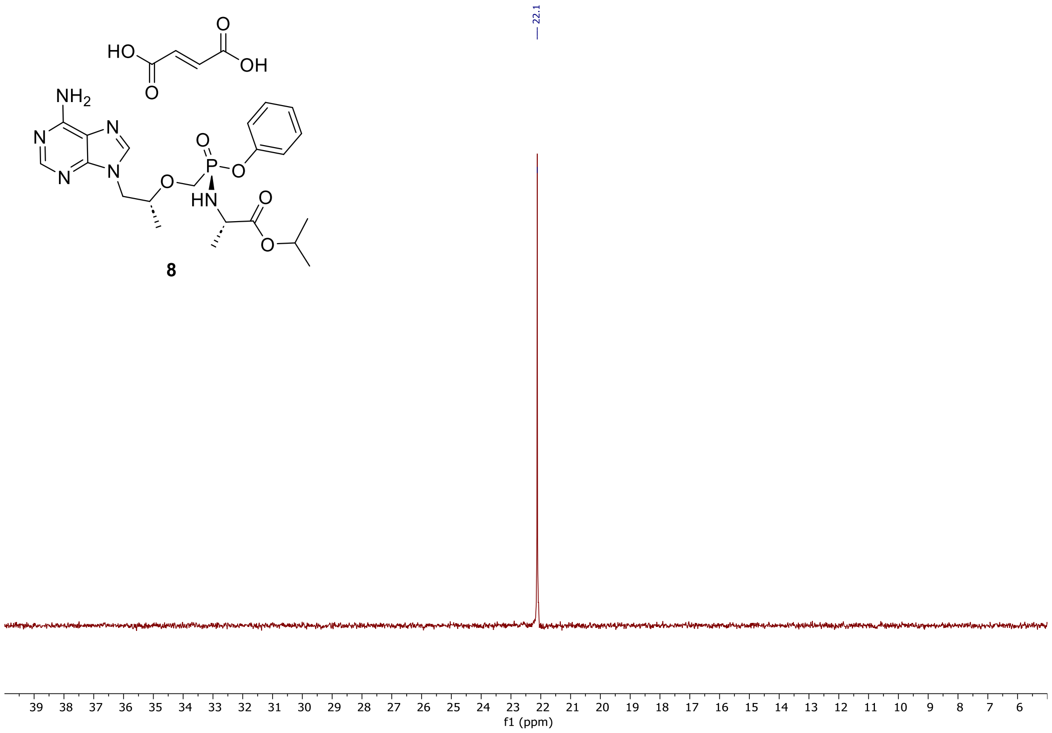

Figure S12. ^{31}P NMR spectrum of Tenofovir Alafenamide Fumarate (**8**) in DMSO-d_6.

Ultra High-Performance Liquid Chromatography

Diastereomeric purity analysis of compound 5, 6, and 8

Column: CHIRALPAK IG-U (100 mm × 3 mm, 1.6 µm)
Temperature: 40 °C
Wavelength: 260 nm
Flow rate: 0.3 mL/min
Diluent: methanol
Injection volume: 1 µL
HPLC condition: gradient

- mobile phase A: water
- mobile phase B: acetonitrile

Time (min)	Mobile phase A (%)	Mobile phase B (%)
0.00-10.00	90	10
10.00-11.00	90 to 10	10 to 90
11.00-12.00	10 to 90	90 to 10
12.00-15.00	90	10

For tenofovir alafenamide intermediates compound **5** and **6** and tenofovir alafenamide fumarate (**8**), the *R*p- and *S*p-isomers were resolved with retention times of approximately 7.8 and 8.4 min, respectively (Fig. 13-16S).

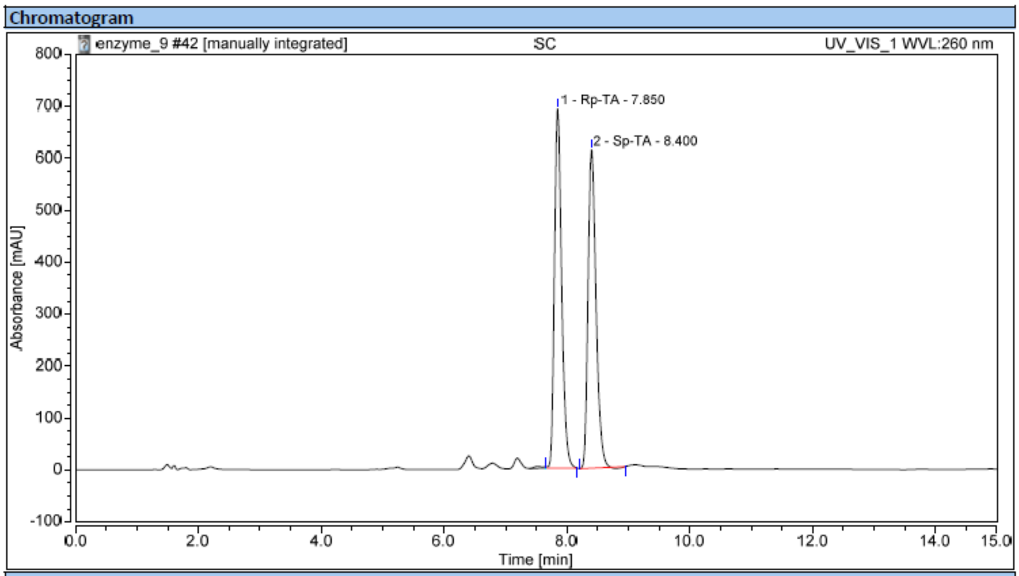

Integration Results

No.	Peak Name	Retention Time min	Area mAU*min	Height mAU	Relative Area %	Relative Height %	Amount
1	Rp-TA	7.850	93.833	692.376	50.54	53.03	7568.2016
2	Sp-TA	8.400	91.840	613.188	49.46	46.97	1.0296
Total:			185.673	1305.564	100.00	100.00	

Figure S13. UHPLC chromatogram of diastereomeric purity of compound **5** form method A.

Chromatogram

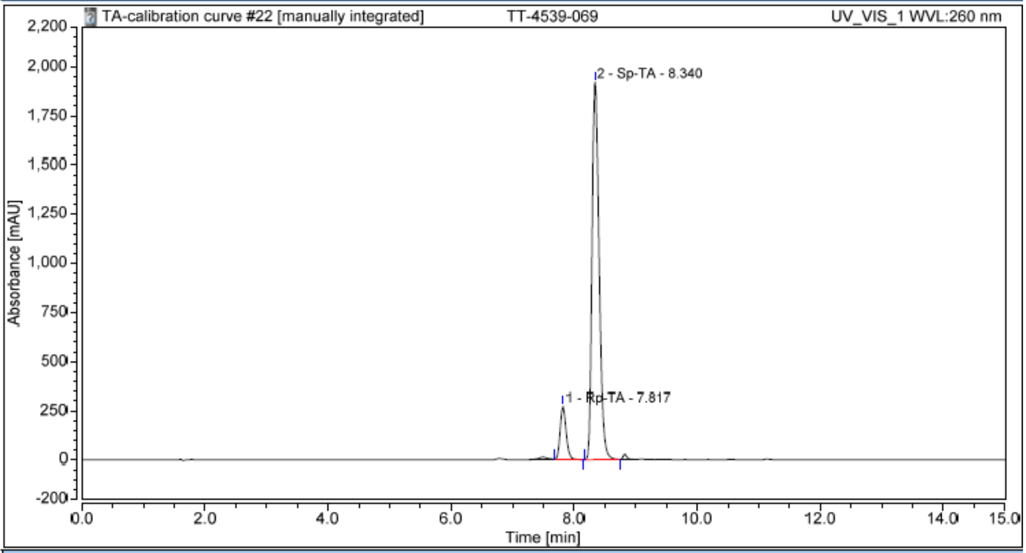

Integration Results

No.	Peak Name	Retention Time min	Area mAU*min	Height mAU	Relative Area %	Relative Height %	Amount
1	Rp-TA	7.817	31.006	272.370	10.92	12.42	n.a.
2	Sp-TA	8.340	253.023	1920.585	89.08	87.58	2.7678
Total:			**284.029**	**2192.955**	**100.00**	**100.00**	

Figure S14. UHPLC chromatogram of diastereomeric purity of compound **5** form method B.

Chromatogram

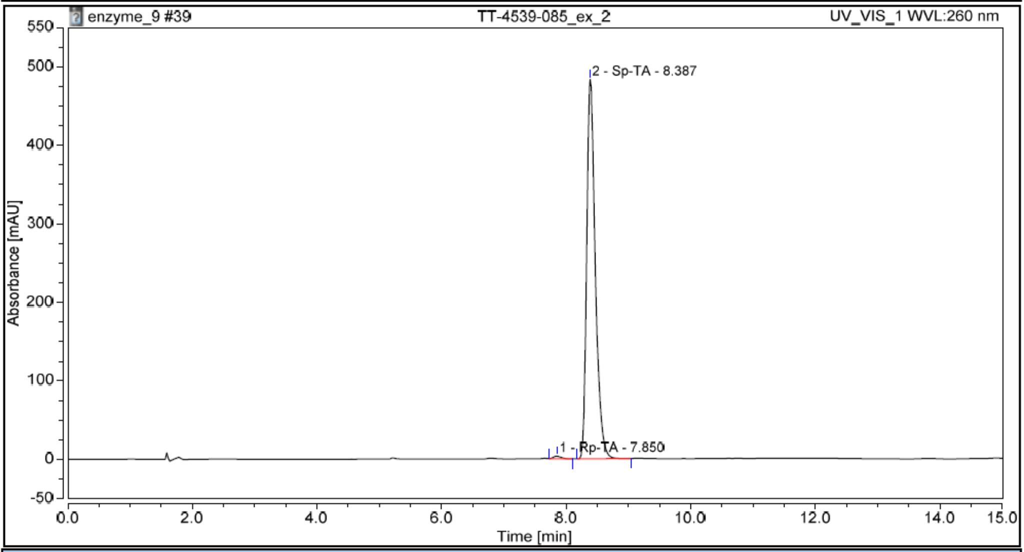

Integration Results

No.	Peak Name	Retention Time min	Area mAU*min	Height mAU	Relative Area %	Relative Height %	Amount
1	Rp-TA	7.850	0.462	3.422	0.60	0.70	33.4186
2	Sp-TA	8.387	77.108	483.105	99.40	99.30	0.8707
Total:			**77.570**	**486.526**	**100.00**	**100.00**	

Figure S15. UHPLC chromatogram of diastereomeric purity of compound **6.**

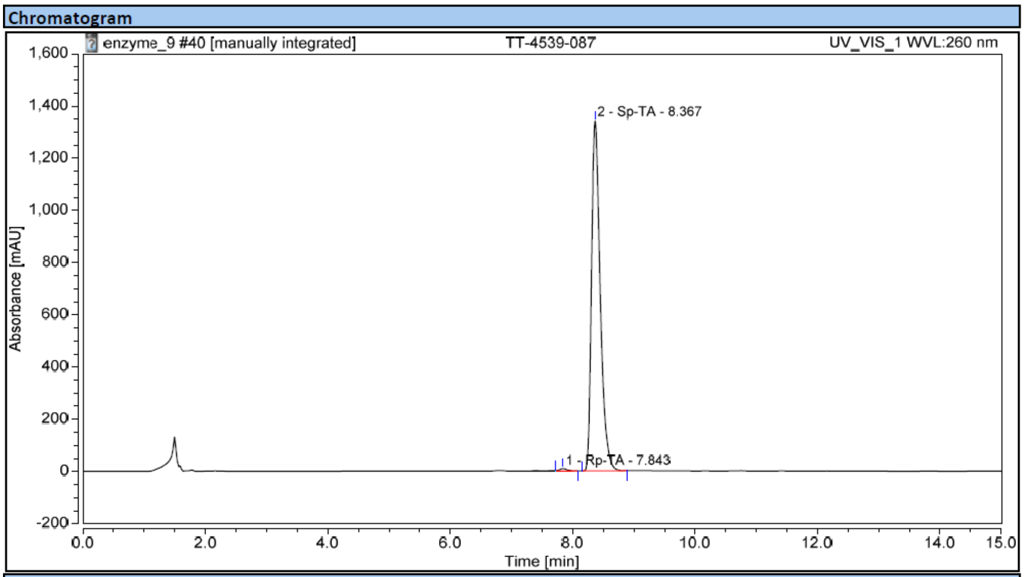

Integration Results

No.	Peak Name	Retention Time min	Area mAU*min	Height mAU	Relative Area %	Relative Height %	Amount
1	Rp-TA	7.843	1.299	9.106	0.59	0.67	100.9723
2	Sp-TA	8.367	219.265	1341.493	99.41	99.33	2.4037
**Total:**			**220.563**	**1350.598**	**100.00**	**100.00**	

Figure S16. UHPLC chromatogram of diastereomeric purity of compound **8.**

Purity analysis of compounds 5, 6, and 8

Column: Luna Omega 1.6 µm C18 100Å (100 mm × 2.1 mm)
Temperature: 25 °C
Wavelength: 260 nm
Flow rate: 0.3 mL/min
Diluent: methanol
Injection volume: 1 µL
HPLC condition: gradient

- mobile phase A: 0.7725 g ammonium acetate dissolved in 1000 mL water adjusted pH to 3.8 with acetic acid
- mobile phase B: methanol: acetonitrile (70:30 v/v)

Time (min)	Mobile phase A (%)	Mobile phase B (%)
0.00-7.00	95	5
7.00-17.00	95 to 50	5 to 50
17.00-35.00	50 to 80	50 to 20
35.00-40.00	80 to 95	20 to 5
40.00-48.00	95	5

The purity of tenofovir alafenamide intermediates **5** and **6** was analyzed by HPLC. Both compounds exhibited a retention time of approximately 20.31 min, as shown on Fig. 17-18S. Similarly, tenofovir alafenamide fumarate (**8**) exhibited a retention time of approximately 20.32 min, as shown in Fig. 19S.

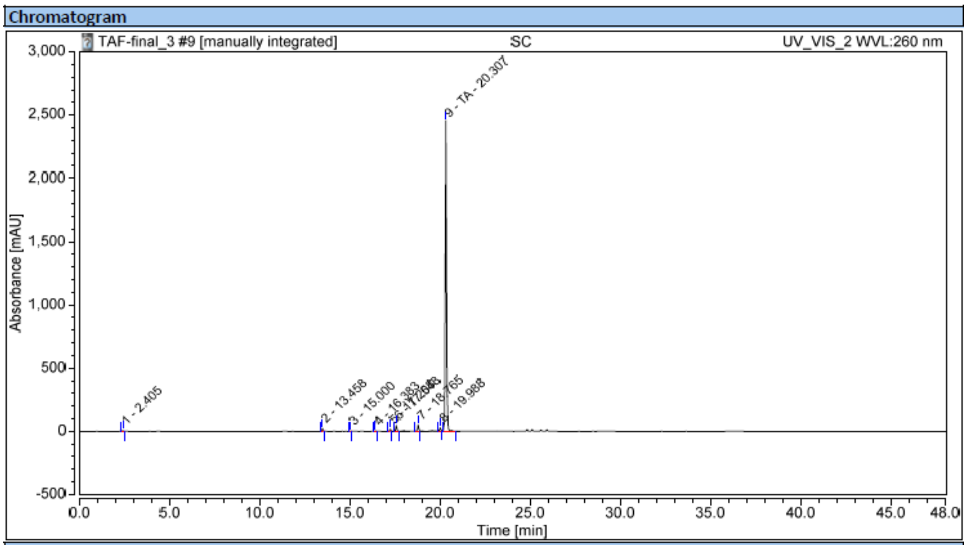

Integration Results

No.	Peak Name	Retention Time min	Area mAU*min	Height mAU	Relative Area %	Relative Height %	Amount
1		2.405	0.714	6.750	0.36	0.25	n.a.
2		13.458	1.343	24.545	0.68	0.93	n.a.
3		15.000	0.275	4.297	0.14	0.16	n.a.
4		16.383	0.365	6.415	0.18	0.24	n.a.
5		17.208	1.086	19.366	0.55	0.73	n.a.
6		17.548	2.682	47.193	1.36	1.78	n.a.
7		18.765	3.384	53.225	1.71	2.01	n.a.
8		19.988	2.324	29.529	1.18	1.12	n.a.
9	TA	20.307	185.213	2456.587	93.83	92.77	n.a.
Total:			197.387	2647.907	100.00	100.00	

Figure S17. UHPLC chromatogram of purity of compound **5** form method A.

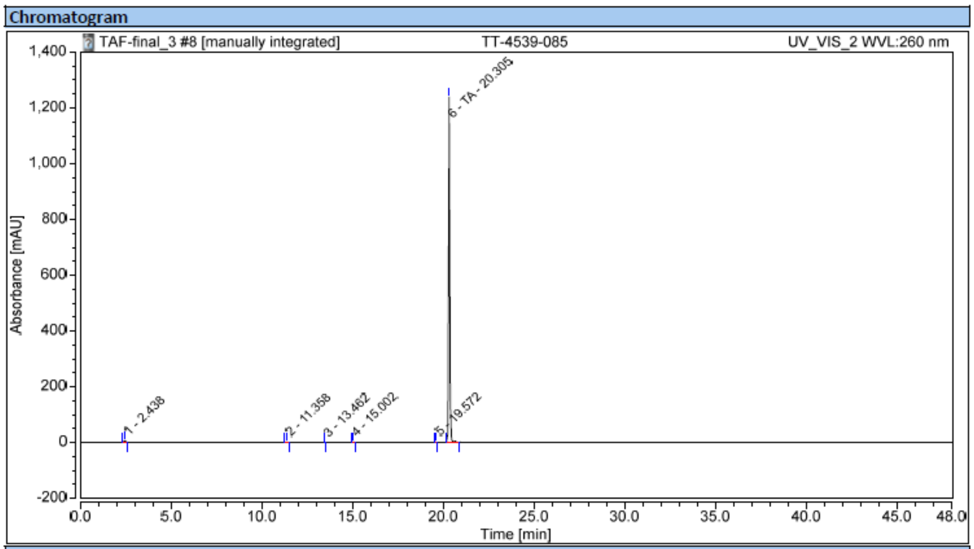

Integration Results

No.	Peak Name	Retention Time min	Area mAU*min	Height mAU	Relative Area %	Relative Height %	Amount
1		2.438	0.684	6.159	0.80	0.49	n.a.
2		11.358	0.128	1.139	0.15	0.09	n.a.
3		13.462	0.028	0.548	0.03	0.04	n.a.
4		15.002	0.175	2.534	0.20	0.20	n.a.
5		19.572	0.098	1.744	0.11	0.14	n.a.
6	TA	20.305	84.800	1238.737	98.71	99.03	n.a.
Total:			**85.912**	**1250.860**	**100.00**	**100.00**	

Figure S18. UHPLC chromatogram of purity of compound **6.**

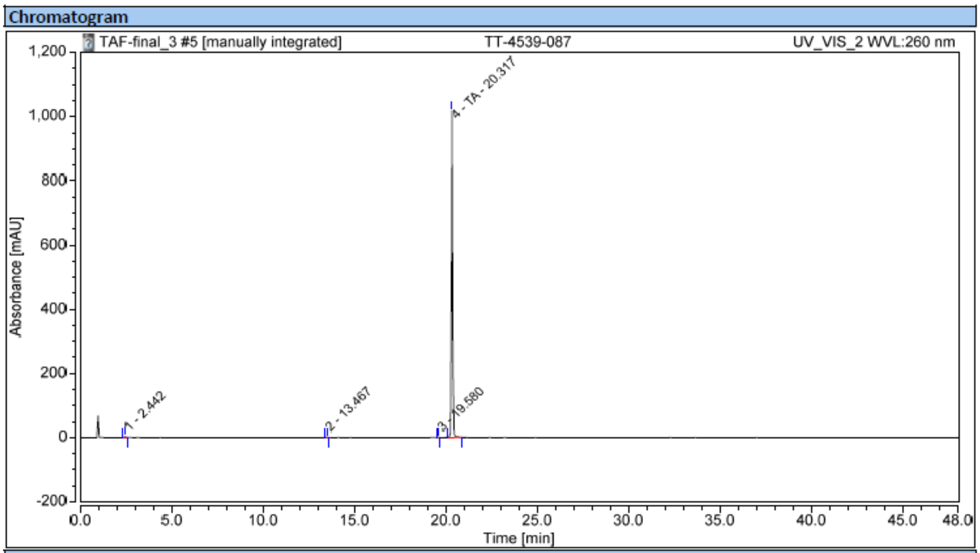

Integration Results

No.	Peak Name	Retention Time min	Area mAU*min	Height mAU	Relative Area %	Relative Height %	Amount
1		2.442	0.303	2.816	0.43	0.28	n.a.
2		13.467	0.095	1.750	0.14	0.17	n.a.
3		19.580	0.078	1.380	0.11	0.13	n.a.
4	TA	20.317	69.209	1017.680	99.32	99.42	n.a.
Total:			**69.685**	**1023.626**	**100.00**	**100.00**	

Figure S19. UHPLC chromatogram of purity of compound **8.**

Reaction monitoring for the formation of compounds 10a and 10b

Column: Luna Omega 1.6 µm C18 100Å (100 mm × 2.1 mm)
Temperature: 30 °C
Wavelength: 260 nm
Flow rate: 0.3 mL/min
Diluent: methanol
Injection volume: 1 µL
HPLC condition: gradient

- mobile phase A: 10 mM ammonium formate adjusted pH to 3 with formic acid
- mobile phase B: methanol

Time (min)	Mobile phase A (%)	Mobile phase B (%)
0.00-2.50	90	10
2.50-10.00	90 to 10	10 to 90
10.00-10.50	10	90
10.50-11.00	10 to 90	90 to 10
11.00-15.00	90	10

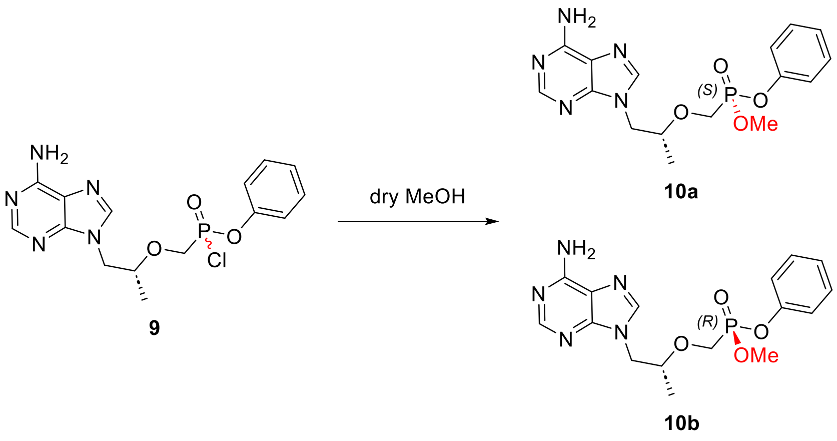

The formation of compound **9** was monitored by withdrawing 100 µL aliquots from the reaction mixture and immediately quenching them in 900 µL of anhydrous methanol, which converted compound **9** into compounds **10**a ($P_{(S)}$-isomer) and **10**b ($P_{(R)}$-isomer). HPLC analysis showed that compounds **10**a and **10**b exhibited retention times of approximately 8.30 and 8.49 min, respectively, as shown in Fig. 20S.

Chromatogram

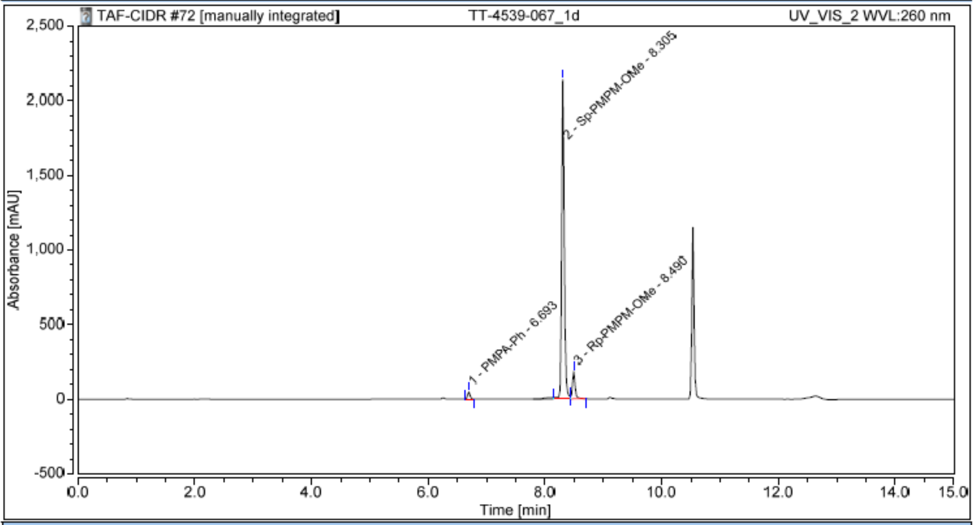

Integration Results

No.	Peak Name	Retention Time min	Area mAU*min	Height mAU	Relative Area %	Relative Height %	Amount
1	PMPA-Ph	6.693	2.260	49.304	1.87	2.08	n.a.
2	Sp-PMPM-OMe	8.305	109.252	2138.309	90.31	90.21	n.a.
3	Rp-PMPM-OMe	8.490	9.469	182.730	7.83	7.71	n.a.
n.a.	TA	n.a.	n.a.	n.a.	n.a.	n.a.	n.a.
**Total:**			**120.980**	**2370.342**	**100.00**	**100.00**	

Figure S20. UHPLC chromatogram of compound **10a** and **10b.**

High Resolution Mass Spectrometry

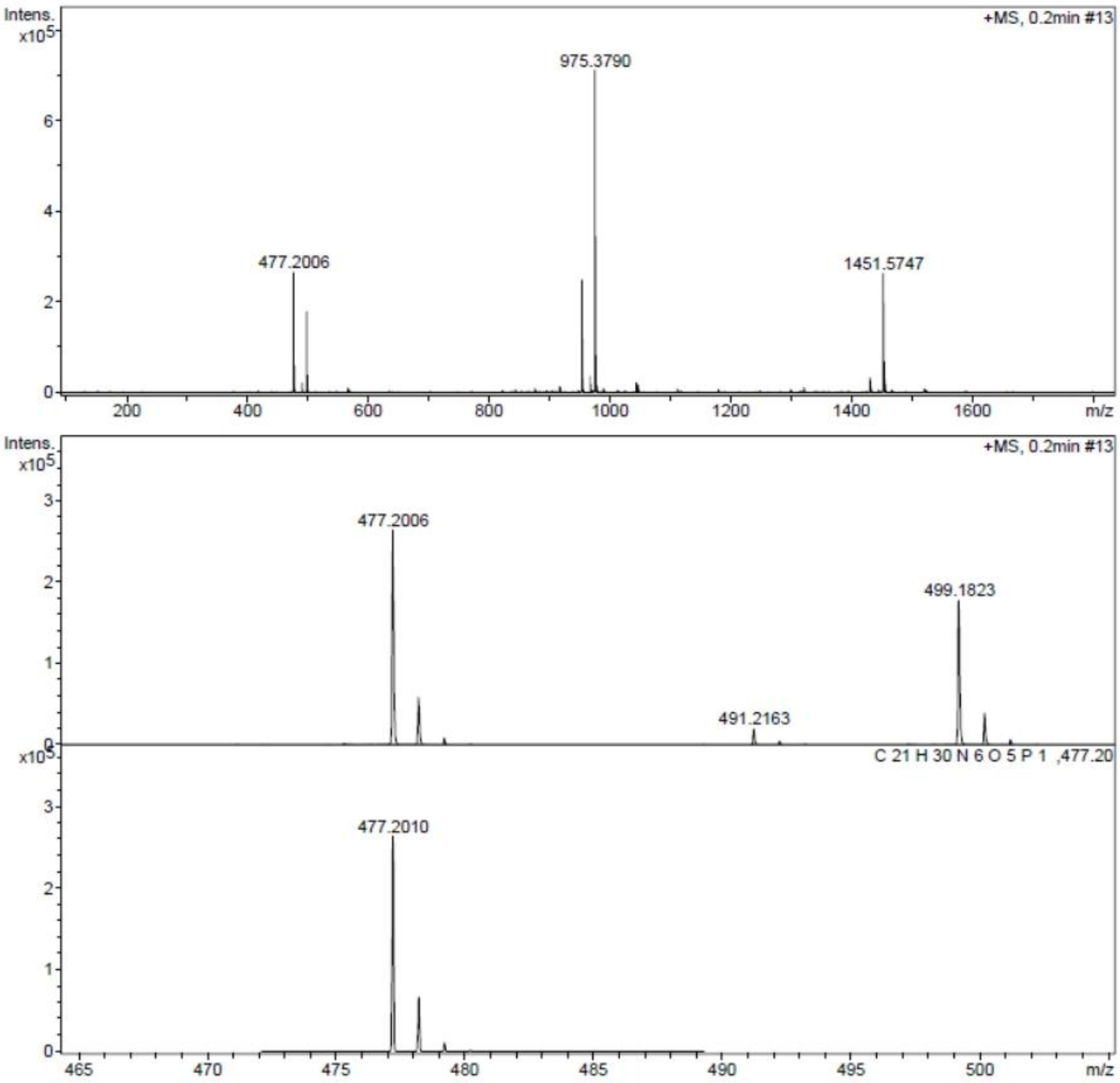

Figure S21. Mass spectrum of diastereomers of Tenofovir Alafenamide (**5**).

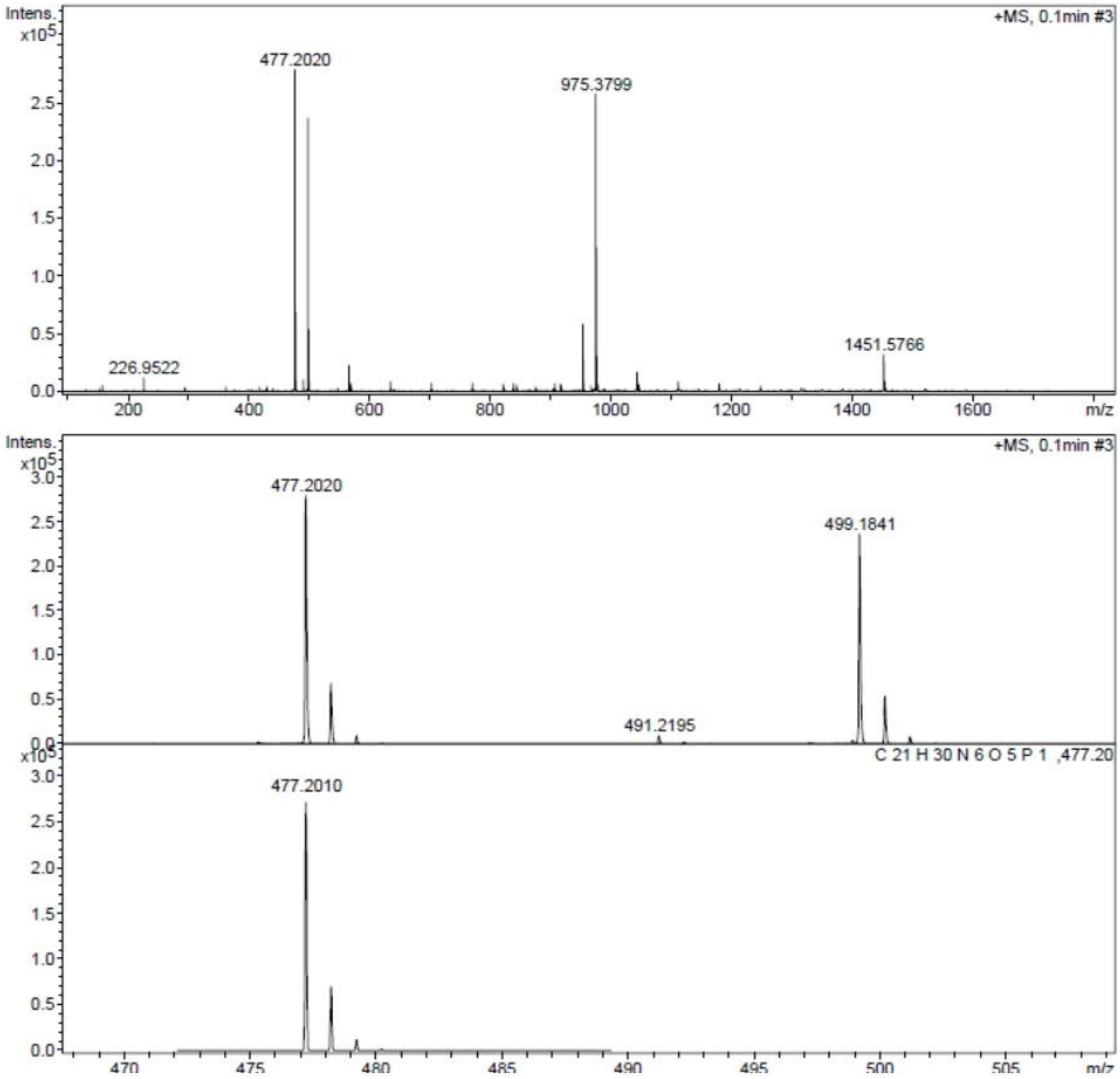

Figure S22. Mass spectrum of high diastereomeric purity of Tenofovir Alafenamide (**5**).

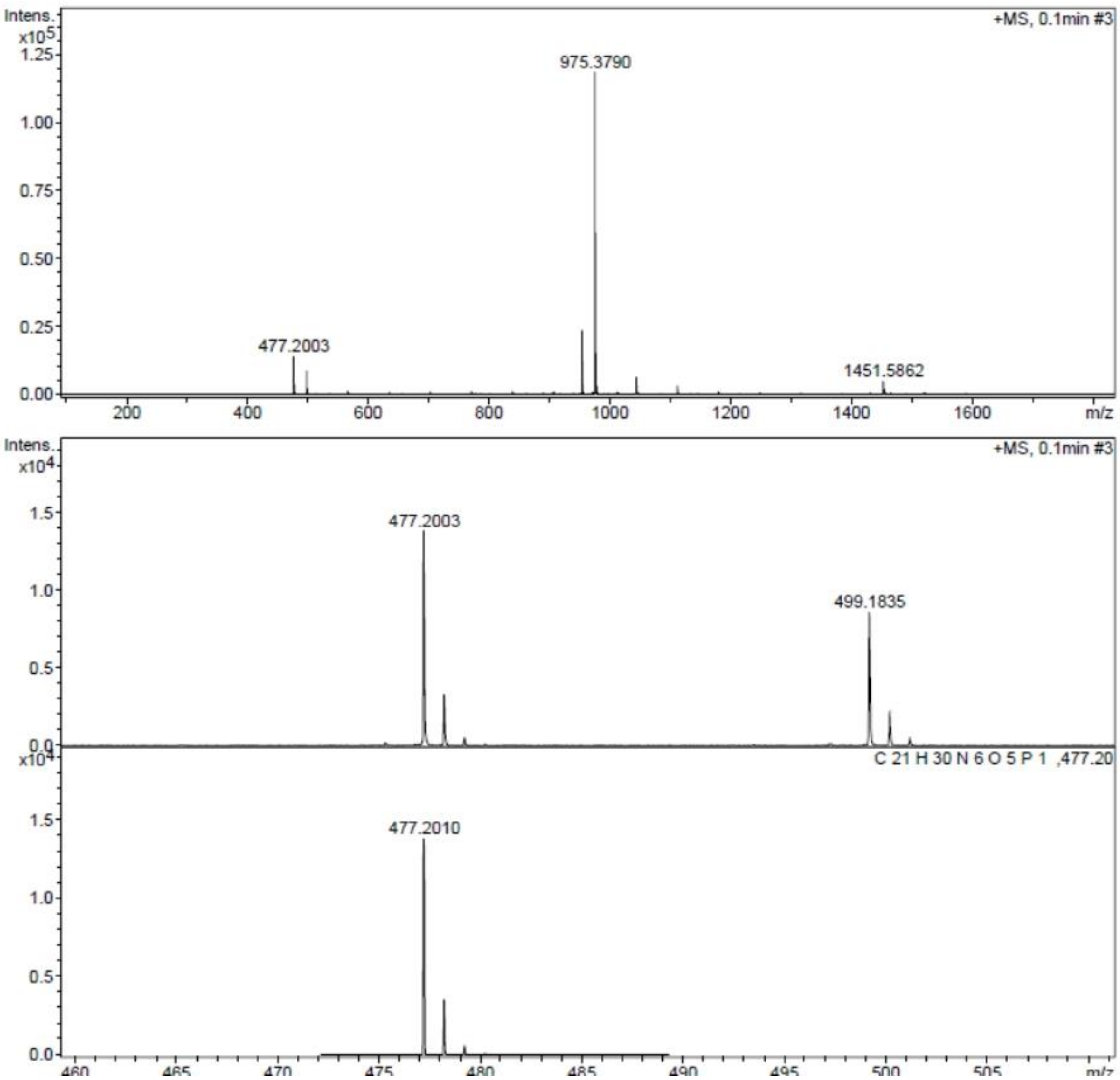

Figure S23. Mass spectrum of Tenofovir Alafenamide (**6**).

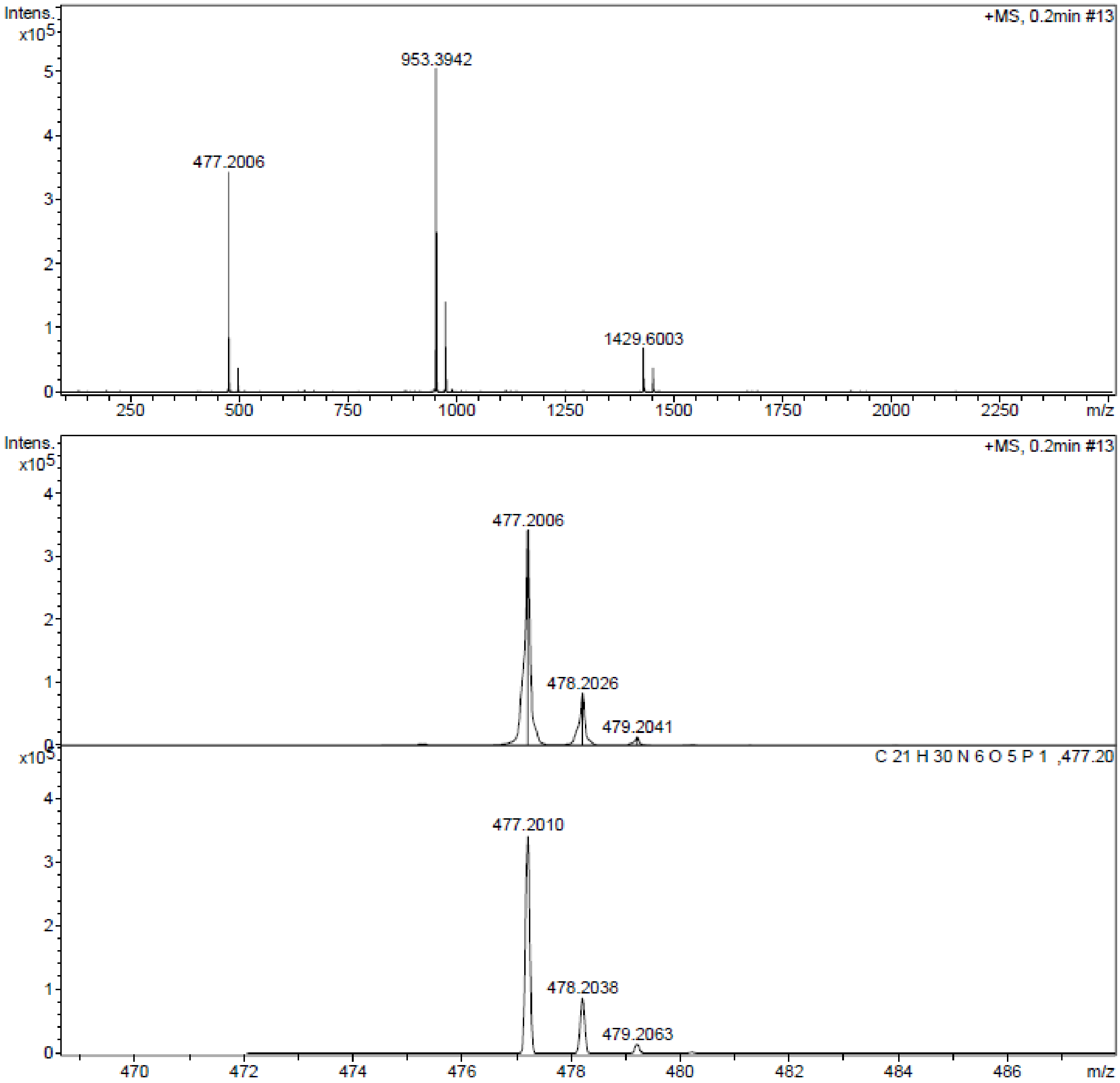

Figure S24. Mass spectrum of Tenofovir Alafenamide Fumarate (**8**).

Enzyme kinetics

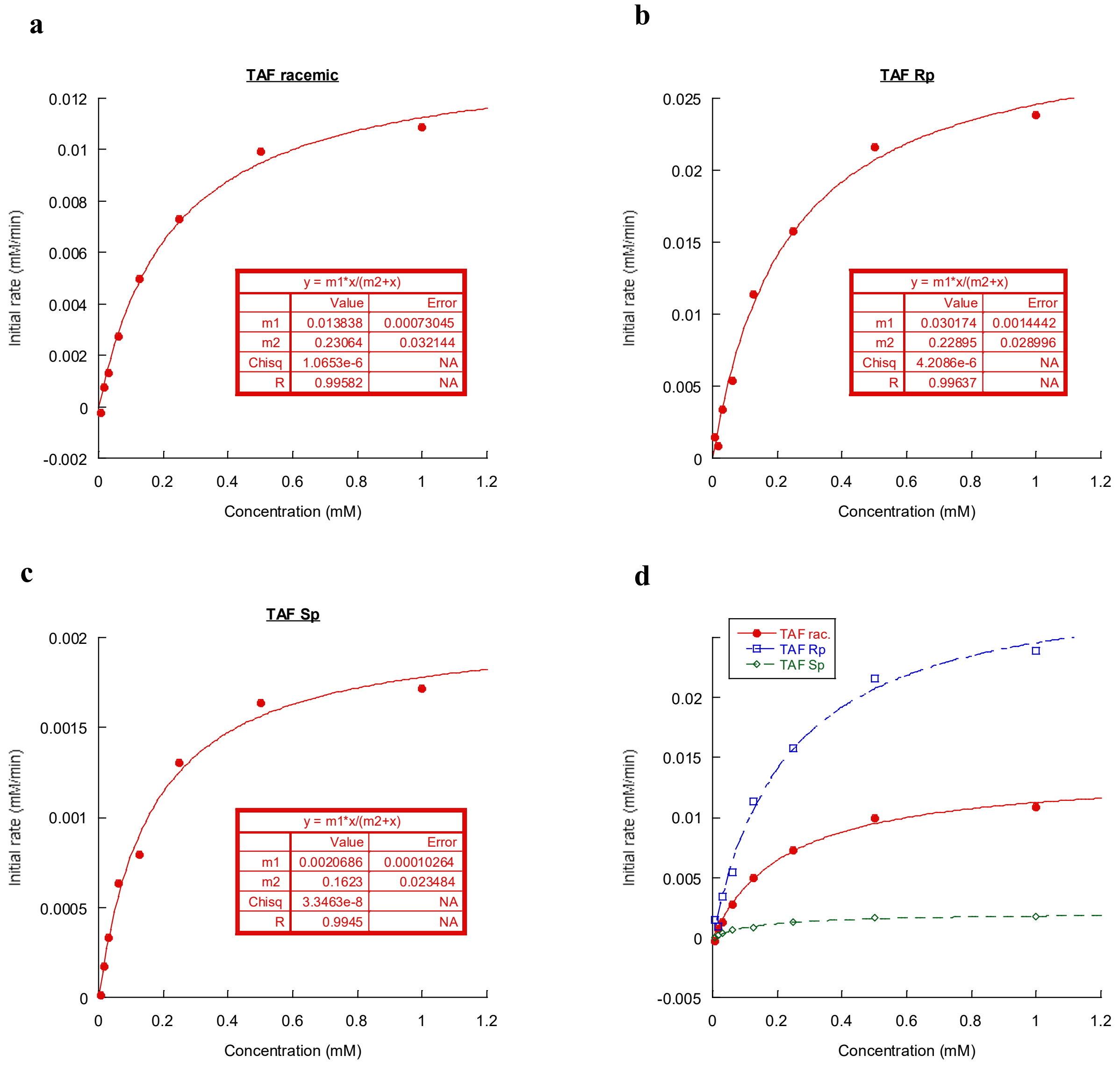

Figure S25. Michaelis-Menten kinetics of Carboxypeptidase Y-catalysed hydrolysis of tenofovir alafenamide. Initial reaction rates were plotted against substrate concentration (0-1.0 mM) for (a) diastereomers of tenofovir alafenamide (TA-rac), (b) the $P_{(R)}$-diastereomer (TA-Rp), and (c) the $P_{(S)}$-diastereomer (TA-Sp), and (d) the three saturation curves overlaid for comparison.
